# A light-driven three-dimensional plasmonic nanosystem that translates molecular motion into reversible chiroptical function

**DOI:** 10.1038/ncomms10591

**Published:** 2016-02-02

**Authors:** Anton Kuzyk, Yangyang Yang, Xiaoyang Duan, Simon Stoll, Alexander O. Govorov, Hiroshi Sugiyama, Masayuki Endo, Na Liu

**Affiliations:** 1 Max Planck Institute for Intelligent Systems, Heisenbergstrasse 3, D-70569 Stuttgart, Germany; 2 Institute for Integrated Cell-Material Sciences (WPI-iCeMS), Kyoto University, Yoshida-ushinomiyacho, Sakyo-ku, Kyoto 606-8501, Japan; 3 Department of Chemistry, Graduate School of Science, Kyoto University, Kitashirakawa-oiwakecho, Sakyo-ku, Kyoto 606-8502, Japan; 4 Kirchhoff Institute for Physics, University of Heidelberg, Im Neuenheimer Feld 227, D-69120 Heidelberg, Germany; 5 Department of Physics and Astronomy, Ohio University, Athens, Ohio 45701, USA

## Abstract

Nature has developed striking light-powered proteins such as bacteriorhodopsin, which can convert light energy into conformational changes for biological functions. Such natural machines are a great source of inspiration for creation of their synthetic analogues. However, synthetic molecular machines typically operate at the nanometre scale or below. Translating controlled operation of individual molecular machines to a larger dimension, for example, to 10–100 nm, which features many practical applications, is highly important but remains challenging. Here we demonstrate a light-driven plasmonic nanosystem that can amplify the molecular motion of azobenzene through the host nanostructure and consequently translate it into reversible chiroptical function with large amplitude modulation. Light is exploited as both energy source and information probe. Our plasmonic nanosystem bears unique features of optical addressability, reversibility and modulability, which are crucial for developing all-optical molecular devices with desired functionalities.

## Abstract (web summary)

Controlled operation of individual molecular machines on a larger scale, 10-100 nm, remains challenging. Here, Kuzyk *et al.* demonstrate a light-driven plasmonic nanosystem that can amplify the molecular motion of azobenzene through a host nanostructure and translates it into reversible chiroptical response.

## Graphical abstract

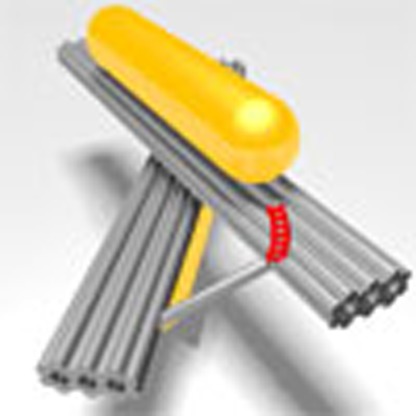

When designing active nanoscale devices, three prerequisites are of paramount importance. First, an efficient energy source for triggering conformation changes at the nanoscale is crucial^1^. Equally important is the reversible control over conformation of individual nanostructures. Last but not least is the ability to report such nanoscale conformation changes and translate them into tunable functionalities^2^,^3^. Among a variety of energy sources, light represents a unique stimulus to power an operation^4^. Different from chemical fuels that unavoidably introduce contaminants in a system, light is clean and waste-free. Also, in contrast to chemical fuels that crucially depend on diffusion kinetics, light offers high spatial and temporal resolution as it can be switched on and off rapidly. Most importantly, light can deliver noninvasive read-out of an optically active system, thus allowing for monitoring an operation in real time.

Here we demonstrate an all-optically controlled plasmonic nanosystem in the visible range using DNA nanotechnology. Our system can amplify the sub-nanometre conformation changes of azobenzene through the active host nanostructure^5^,^6^ and consequently translate the light-induced molecular motion of azobenzene into reversible plasmonic chiroptical response, which can be *in situ* read out by optical spectroscopy. The plasmonic nanostructure comprises two gold nanorods (AuNRs) assembled on a reconfigurable DNA origami template^7^,^8^,^9^,^10^,^11^,^12^,^13^,^14^. A photoresponsive active site is introduced on the template with an azobenzene-modified DNA segment^15^. Light can cyclically ‘write' and ‘erase' the conformation states of the nanostructure through photoisomerization of azobenzene at a localized region. Different conformation states are read by probe light.

## Results

### Design of the photoresponsive nanostructures

**Figure 1:**
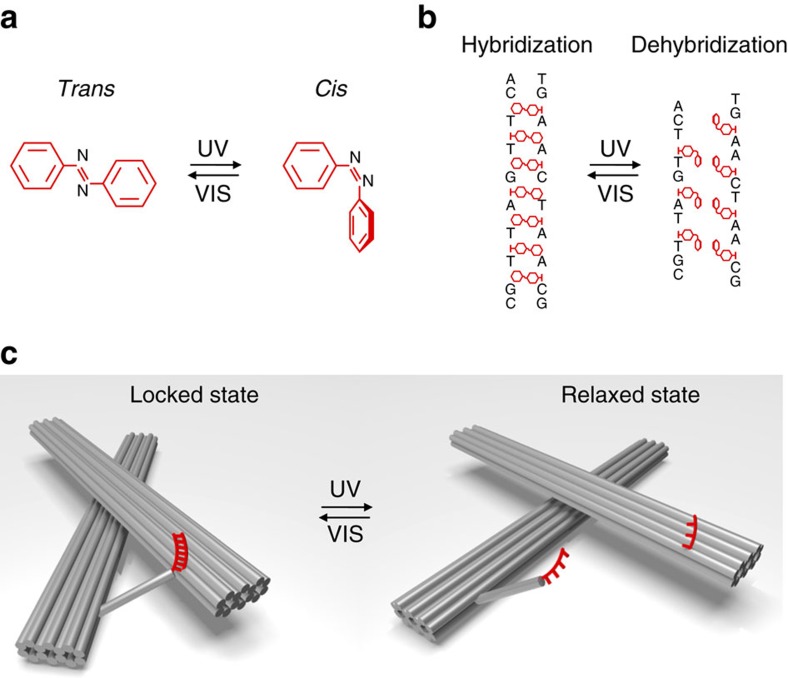
Light-induced conformation changes of DNA origami nanostructures. (**a**) *Trans*–*cis* photoisomerization of an azobenzene molecule by ultraviolet (UV) and visible (VIS) light illumination. (**b**) Hybridization and dehybridization of azobenzene-modified DNA oligonucleotides controlled by *trans*–*cis* photoisomerization of azobenzene through UV and VIS light illumination. (**c**) Photoregulation of the DNA origami template between the locked and relaxed states by UV and VIS light illumination. The active function of the origami structure is enabled by introducing the azobenzene-modified DNA segment (red) in **b** on the template, which works as a recognition site to receive light stimuli for triggering light-induced motion.

Photoisomerization of azobenzene^16^ (Fig. 1) is widely used for the construction of light-driven artificial molecular machines. In particular, azobenzene can be incorporated into DNA strands for reversible control of DNA hybridization^15^,^17^,^18^,^19^ (Fig. 1b). Our active nanostructure is based on a three-dimensional (3D) reconfigurable DNA origami template (Fig. 1c), which consists of two 14-helix bundles (80 nm × 16 nm × 8 nm), folded from a long single-stranded DNA scaffold, with the help of hundreds of staple strands (Supplementary Methods, Supplementary Tables 1 and 2 and Supplementary Figs 1–5). The two linked origami bundles form a chiral object with a tunable angle^14^ (Supplementary Fig. 2). The active function of the structure is enabled by introducing an azobenzene-modified DNA segment on the template, which works as a recognition site to receive light stimuli. This photoresponsive segment comprises two DNA branches, which are extended from the two origami bundles, respectively. One branch possesses a double-stranded DNA (dsDNA) 20-base-pair part linked by disulfide bonds with azobenzene-modified oligonucleotides (Azo-ODN 1)^18^,^19^. The other branch contains Azo-ODN 2, which is pseudocomplementary to Azo-ODN 1. Azo-ODN 1 and Azo-ODN 2 contain three and four azobenzene modifications, respectively (Supplementary Methods and Supplementary Fig. 3). Multiple azobenzene modifications are essential for efficient photoregulation of DNA hybridization^17^,^20^. On ultraviolet light illumination, the azobenzene molecules in Azo-ODNs are converted to *cis*-form, resulting in dehybridization of the Azo-ODN duplex. The photoresponsive segment is opened and the conformation of the origami nanostructure is therefore ‘relaxed'. In contrast, on visible light illumination, the azobenzene molecules are converted to *trans*-form and Azo-ODNs can be hybridized into the Azo-ODN duplex. Therefore, the photoresponsive segment is locked. The dsDNA part is employed here to define a rigid angle between the two origami bundles for a stable chiral conformation.

### Photoregulation of the DNA origami templates

**Figure 2:**
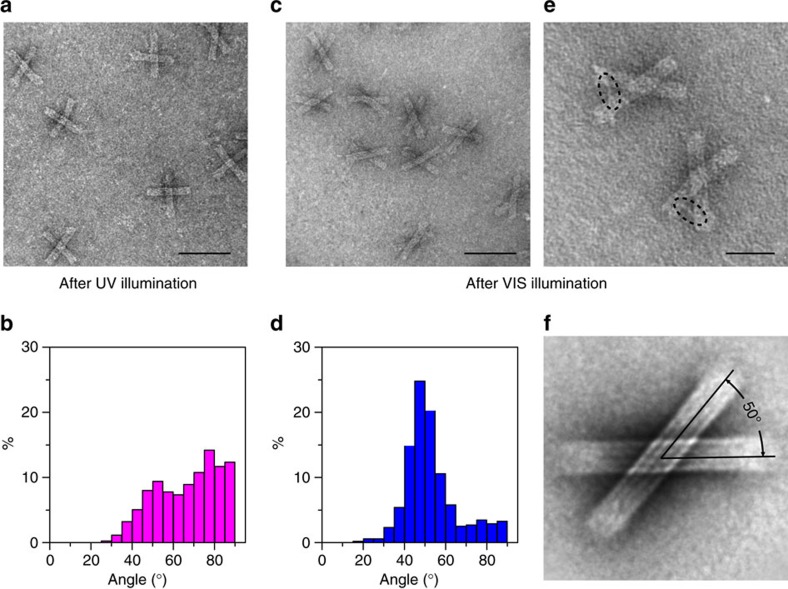
Structural characterization of the DNA origami nanostructures. (**a**) TEM image of the DNA origami nanostructures after ultraviolet (UV) light illumination. (**b**) Statistic histogram of the acute angle between two linked origami bundles after UV light illumination. The number of the analysed structures: 463. A broad distribution over angles is observed. (**c**) TEM image of the DNA origami nanostructures after visible (VIS) light illumination. The locked state is designed to be right-handed. (**d**) Statistic histogram of the acute angle between two linked origami bundles after VIS light illumination. The number of the analysed structures: 541. A maximum magnitude over angles occurs around 50°, which is in accordance to our structure design. (**e**) Enlarged view of the origami structures in the locked state. The dsDNA branch, which links the two origami bundles to define the angle, is clearly visible. (**f**) Averaged TEM image reconstructed from locked origami structures. It evidently demonstrates the excellent structural homogeneity and high angle accuracy within the locked structures. Scale bars, 100 nm (**a**,**c**); 50 nm (**e**).

It has been reported that the illumination time and temperature affect the hybridization and dehybridization kinetics of the Azo-ODN duplex^18^,^20^. To ensure good switching efficiency and simultaneously avoid origami damage, the sample was kept at a temperature of 40 °C during all switching experiments^18^. As a representative case, the locked state of the origami template was with a right-handed conformation, in which the angle between the two bundles was ∼50°. The sample was first illuminated by ultraviolet light (365 nm) for 15 min and then by visible light (450 nm) for 10 min. Transmission electron microscopy (TEM) images of the sample after ultraviolet and visible light illumination are shown in Fig. 2a,c, respectively (for additional TEM images see Supplementary Figs 5–11. Statistic histograms of the acute angle between two linked origami bundles based on an assessment of ∼400 origami structures after ultraviolet and visible light illumination are presented in Fig. 2b,d, respectively, and Supplementary Fig. 12. As shown in Fig. 2b, after ultraviolet light illumination, a broad distribution over angles is observed, with a maximum magnitude occurring around 90°. This reveals that the origami structures have been turned into the relaxed state by ultraviolet light. Here 90° is more favourable owing to the electrostatic repulsion between the two bundles within one origami structure. On the other hand, after visible light illumination, a maximum magnitude over angles occurs around 50°, which is in accordance with our structure design (Supplementary Fig. 2). This elucidates that a majority of the origami structures have been driven by visible light to the designated locked state. An enlarged view of the origami structures in the locked state is shown in Fig. 2e. The dsDNA branch, which links the two origami bundles to define the angle, is clearly visible in the individual structures in Fig. 2e. An averaged TEM image reconstructed from the perfectly locked origami structures (∼120) is presented in Fig. 2f. It demonstrates the excellent structural homogeneity and high angle accuracy within the locked structures. The TEM characterization reveals that *trans*–*cis* photoisomerization of azobenzene, which is associated with a molecular length change of∼3.5 Å (ref. 21) can be efficiently amplified by the origami structures into their distinct conformation changes on the order of 30 nm (Supplementary Fig. 2). This corresponds to an amplification factor of ∼100. Certainly, this amplification factor can be further increased by designing larger origami frames or larger angle changes. Moreover, given the remarkable precision of addressability afforded by DNA, this translation can be well controlled in an individual nanostructure at a localized region, which serves as an active recognition site in response to light stimuli.

### Light-driven 3D plasmonic nanosystem

**Figure 3:**
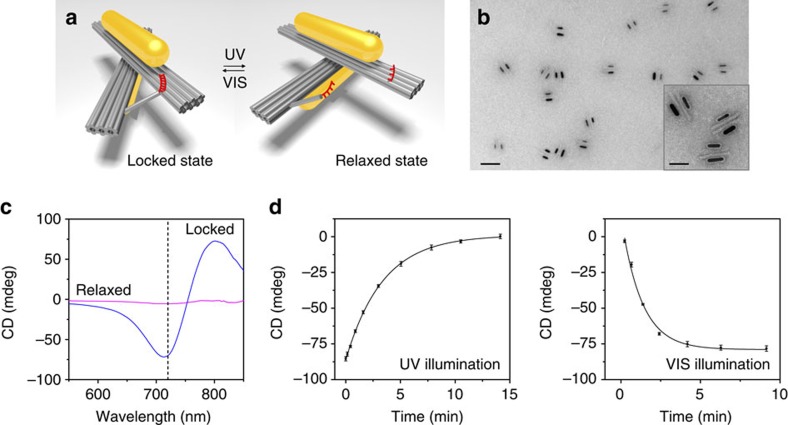
Light-driven 3D plasmonic nanosystem. (**a**) Schematic of the 3D plasmonic nanosystem regulated by ultraviolet (UV) and visible (VIS) light illumination for switching between the locked right-handed and relaxed states. Two AuNRs are assembled on one origami template to form a 3D plasmonic chiral nanostructure. (**b**) TEM images of the plasmonic nanostructures in the locked right-handed state. Scale bars, 200 and 50 nm in the large image and in the inset image, respectively. (**c**) Measured CD spectra after UV (purple) and VIS (blue) illumination. (**d**) Kinetic characterization of the 3D plasmonic nanostructures switching from the locked right-handed state to the relaxed state and vice versa on UV and VIS illumination. The experimental data can be well fit by first-order reaction kinetics with rate constants of 5 × 10^−3^ and 1.3 × 10^−2^ s^−1^ for UV and VIS illumination, respectively. The error bars represent one s.d. from the mean.

Positioning of plasmonic nanoparticles with high precision offered by DNA^22^,^23^,^24^,^25^,^26^,^27^,^28^,^29^,^30^ further endows our light-driven systems with unique optical functionalities. To this end, two AuNRs are assembled on one origami template to form a 3D plasmonic chiral nanostructure (Fig. 3a). Twelve binding sites on each origami bundle are extended with capture strands for robust assembly of one AuNR (38 nm × 10 nm) functionalized with DNA complementary to the capture strands. The length of the binding site area is ∼36 nm (Supplementary Fig. 2). To ensure a high positioning accuracy of the AuNRs on origami, an additional thermal annealing procedure was carried out. Detailed information on the AuNR functionalization and assembly can be found in Supplementary Methods and Supplementary Fig. 13.

When light interacts with the 3D chiral nanostructure, plasmons are excited in the two AuNRs that are placed in close proximity. The excited plasmons are collectively coupled in the cross conformation, leading to plasmonic chiroptical response^31^,^32^,^33^,^34^,^35^. The resulting plasmonic circular dichroism (CD)^36^ spectra are very sensitive on conformation changes, ideal for optically monitoring the conformation evolution in real time^14^.

Figure 3b and Supplementary Fig. 14 show TEM images of the plasmonic nanostructures. A high assembly yield of the AuNR dimers on the origami templates has been achieved. Owing to a higher affinity of the AuNRs to the carbon film of the TEM grid compared with that of DNA, the AuNR pairs appear side by side in the TEM images. To *in situ* monitor the dynamic process associated with the conformation changes triggered by light, the CD response of a plasmonic sample was measured during visible (450 nm) and ultraviolet (365 nm) light illumination, respectively, using a J-815 CD spectrometer (Jasco). To be more specific, visible and ultraviolet light is used to ‘write' and ‘erase' the handed state of the plasmonic system, respectively, while circularly polarized light is used to ‘read' the state changes. For a better elucidation, two representative CD spectra recorded after the system has achieved stable states for visible and ultraviolet light illumination are presented in Fig. 3c. The spectra were recorded within a wavelength range of 550–850 nm. The CD spectrum after visible light illumination is characterized by a bisignate dip-to-peak profile (in blue), which is typical for a right-handed system. This demonstrates that visible light has successfully driven the plasmonic system to the locked state, in which the conformation is ‘written' as right-handed. On the other hand, the CD response after ultraviolet light illumination decreases significantly as shown by the purple curve. The plasmonic system has been converted into the relaxed state and the previous right-handed conformation is therefore ‘erased'. A CD intensity modulation as high as 10 times between the two states has been achieved, demonstrating excellent photoresponsivity of the active plasmonic system. In this regard, molecular motion of azobenzene is spatially amplified by the host nanostructures and optically reflected through distinct chiroptical response changes. For the additional details of optical characterization, see Supplementary Methods and Supplementary Figs 15 and 16.

To provide deeper insight, theoretical calculations were performed using the commercial software COMSOL Multiphysics based on a finite element method (Supplementary Methods), and the results are shown in Supplementary Fig. 17. The CD spectra were calculated as the difference of extinction for the left- and right-handed circularly polarized light. The assembled nanostructures were randomly dispersed in solution, and therefore averaging over different orientations was carried out. To account for the inhomogeneous spectral broadening resulting from the polydispersity of the AuNRs, the dielectric function of Au was modified by including an extra damping coefficient. Overall, the agreement between the experiment and theory is good.

**Figure 4:**
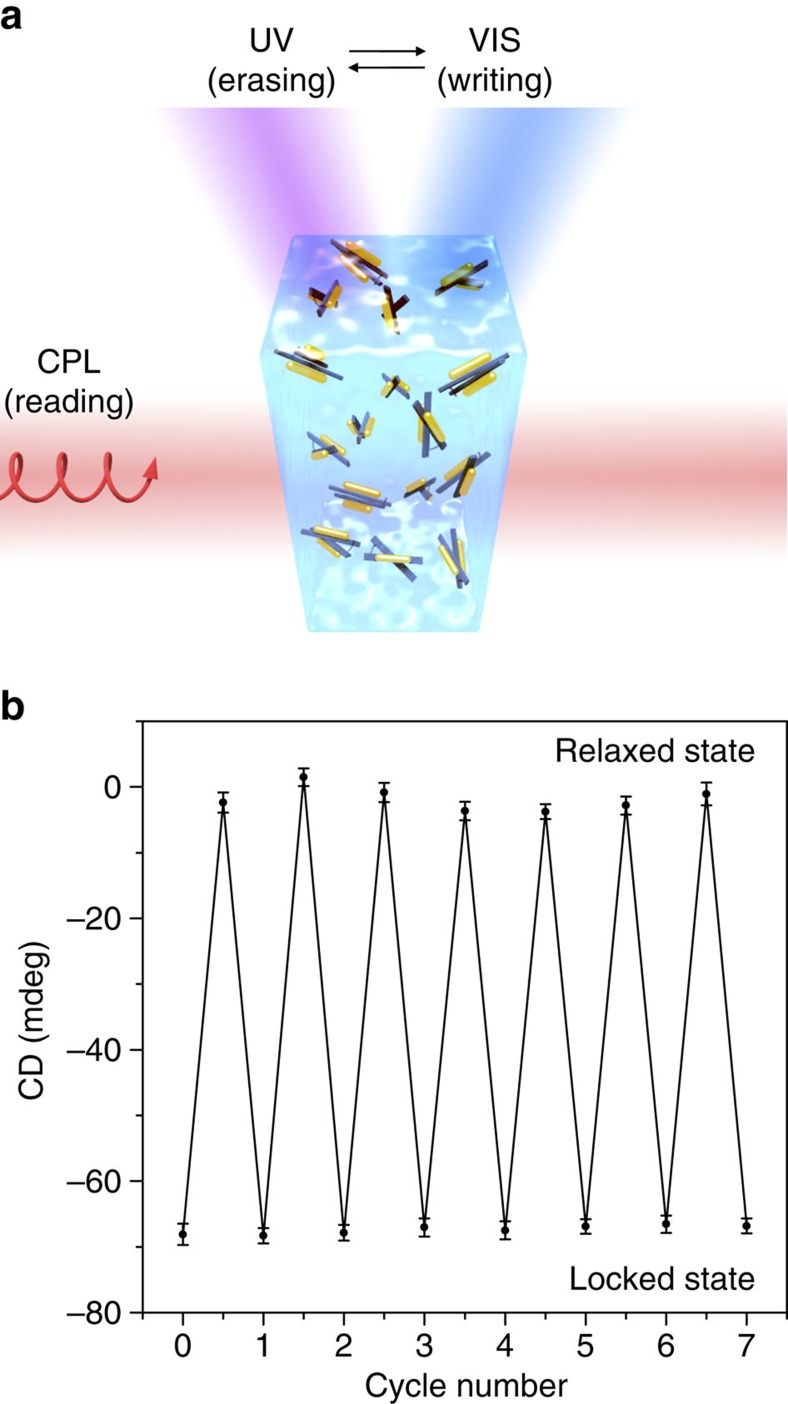
‘Writing', ‘erasing' and ‘reading' of the 3D plasmonic nanostructures by light. (**a**) Reversible conversion of the plasmonic nanostructures between the relaxed and locked right-handed states by ultraviolet (UV) and visible (VIS) light illumination, which performs the ‘erasing' and ‘writing' behaviour, respectively. The resulting conformation states are probed by circularly polarized light (CPL) in real time, which performs the ‘reading' behaviour. (**b**) CD intensity recorded at 720 nm (Fig. 3c) during alternative UV and VIS illumination in multiple cycles. Excellent reversibility of the chiroptical response is achieved between the two states with large signal modulations. The error bars represent one s.d. from the mean.

Next, the CD intensities at 720 nm, that is, approximately at the spectral dip position, are presented as a function of visible and ultraviolet light illumination time in Fig. 3d. The conversion from the right-handed state on ultraviolet light illumination took ∼15 min to achieve the stable relaxed state, whereas the conversion from the relaxed state on visible light illumination took ∼10 min to reach the stable right-handed state. The data curves can be well fit by first-order reaction kinetics with rate constants of 5 × 10^−3^ and 1.3 × 10^−2^ s^−1^ for ultraviolet and visible illumination, respectively. Also, the reversibility of the conversion is examined by alternative ultraviolet and visible light illumination in cycles for 15 and 10 min per exposure, respectively (Fig. 4a and Supplementary Fig. 16). The CD intensity was recorded at 720 nm. As shown in Fig. 4b, excellent reversibility of the chiroptical response is achieved between the two states with large signal modulations. In brief, ‘writing', ‘erasing' and ‘reading' actions can be coordinated efficiently with such a bistable system, in which each state can be converted into the other by light and consequently be reported by light.

## Discussion

The realization of light-driven plasmonic systems based on DNA nanotechnology offers many advantages for effectively manipulating materials and information at the nanoscale. From the material aspect, DNA as one of the most flexible materials in nanotechnology possesses unique biochemical specificity, remarkable spatial accuracy and ease of addressability^37^,^38^,^39^. Inclusion of chemical species that can execute reversible transformations by light such as azobenzene endows such hybrid systems with both spatial and temporal precision. From the information aspect, light as a stimulus renders ‘writing' and ‘erasing' of the conformation states in a reversible way possible without addition of any reagent. Meanwhile, light also serves as an information probe to read dynamic state changes in real time. Our plasmonic system may launch a new generation of sensing platforms^40^,^41^, as light-induced structural changes could be optically tracked and successively retrieved. Finally, by harvesting light energy, individual nanostructures may generate collective actions and directly translate controlled molecular motion to a macroscopic level^42^.

## Methods

### Materials

DNA scaffold strands (p7650) were purchased from Tilibit Nanosystems. Unmodified staple strands (purification: desalting) were purchased from Eurofins MWG. Capture strands for the AuNRs (purification: desalting) were purchased from Sigma-Aldrich. Thiol-modified strands (purification: HPLC) were purchased from http://www.biomers.net/. Azobenzene-modified DNA strands were obtained following a previously published procedure^18^. Agarose for electrophoresis and SYBR Gold nucleic acid stain were purchased from Life Technologies. Uranyl formate for negative TEM staining was purchased from Polysciences, Inc. AuNRs were purchased from Sigma-Aldrich (catalogue no. 716812). Other chemicals were purchased either from Carl-Roth or from Sigma-Aldrich.

### Design and preparation of the DNA origami templates

The design of the DNA origami structures was adopted from a previous study^14^. The strand routing diagram of the origami structures can be found in Supplementary Fig. 1. The sequences of the staple strands and modifications used for photoswitching are provided in Supplementary Table 1. The origami structures were prepared by thermal annealing (Supplementary Table 2) and purified by agarose gel electrophoresis (Supplementary Fig. 4) For details, see Supplementary Methods.

### Light-driven conformational switching

For ultraviolet illumination, a 3-W light-emitting diode (Köhler Technologie-Systeme GmbH & Co. KG) with emission wavelength centred at 365 nm (±5 nm) was used. For visible light illumination, a white light-emitting diode (M7RX, LED LENSER) and a bandpass optical filter centred at 450 nm with a 40 nm bandpass region (FB450-40, Thorlabs) were used. For TEM characterizations, the DNA origami sample was incubated at 40 °C and pH 8. First, the origami sample was exposed to ultraviolet light illumination for 15 min, and part of the sample was used for TEM investigations. The rest of the sample was exposed to visible light illumination for 10 min and then used for subsequent TEM investigations.

### TEM characterization

The DNA origami structures (with or without AuNRs) were imaged using a Philips CM 200 TEM operating at 200 kV. For imaging, the DNA origami structures (with or without AuNRs) were deposited on freshly glow-discharged carbon/formvar TEM grids. The TEM grids were treated with a uranyl formate solution (0.75%) for negative staining of the DNA structures. Angles between the origami bundles in individual structures were obtained by manual analysis of the TEM images. The acute angles were chosen for analysis. Class average images (Fig. 2f) were obtained using EMAN2 software^43^.

### Optical characterizations

CD and ultraviolet–visible measurements were performed with a J-815 Circular Dichroism Spectrometer (Jasco) using Quartz SUPRASIL cuvettes (105.203-QS, Hellma Analytics) with a path length of 10 mm. ultraviolet–visible measurements were also performed with a BioSpectrometer (Eppendorf). Technical details can be found in Supplementary Methods.

## Additional information

**How to cite this article:** Kuzyk, A. *et al.* A light-driven three-dimensional plasmonic nanosystem that translates molecular motion into reversible chiroptical function. *Nat. Commun.* 7:10591 doi: 10.1038/ncomms10591 (2016).

## Supplementary Material

Supplementary Information Supplementary Figures 1-17, Supplementary Tables 1-2, Supplementary Methods and Supplementary References
