# Supplementary material for: A light-driven three-dimensional plasmonic nanosystem that translates molecular motion into reversible chiroptical function
Source: Nat Commun. 2016 Feb 2;7:10591. doi: 10.1038/ncomms10591 (PMC4740900; doi:10.1038/ncomms10591)
Supplement: Supplementary Information — Supplementary Figures 1-17, Supplementary Tables 1-2, Supplementary Methods and Supplementary References [file ncomms10591-s1.pdf]

Supplementary Information

A light-driven three-dimensional plasmonic nanosystem that translates molecular motion into reversible chiroptical functions

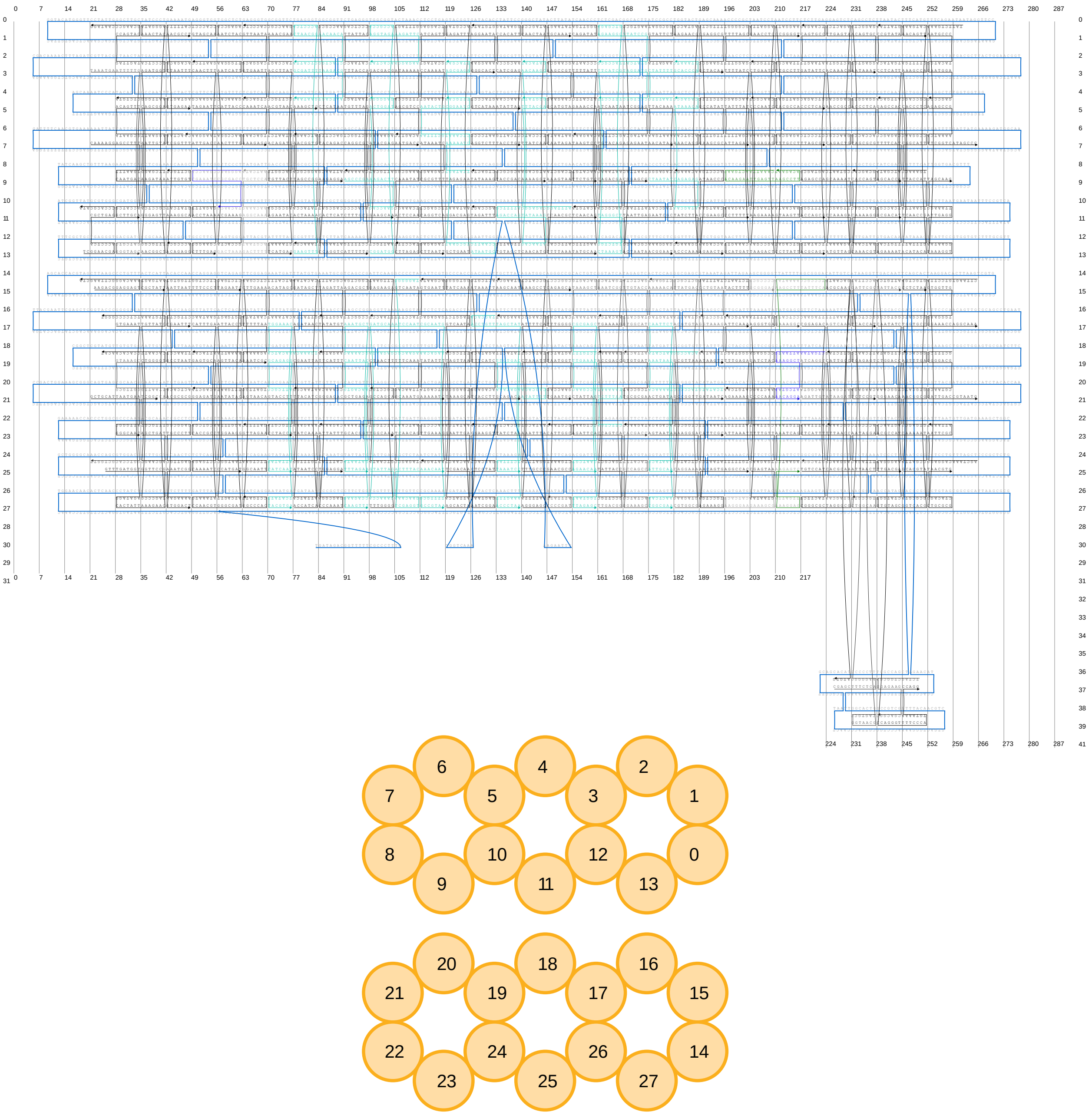

Supplementary Figure 1. Strand routing diagram of the DNA origami template.

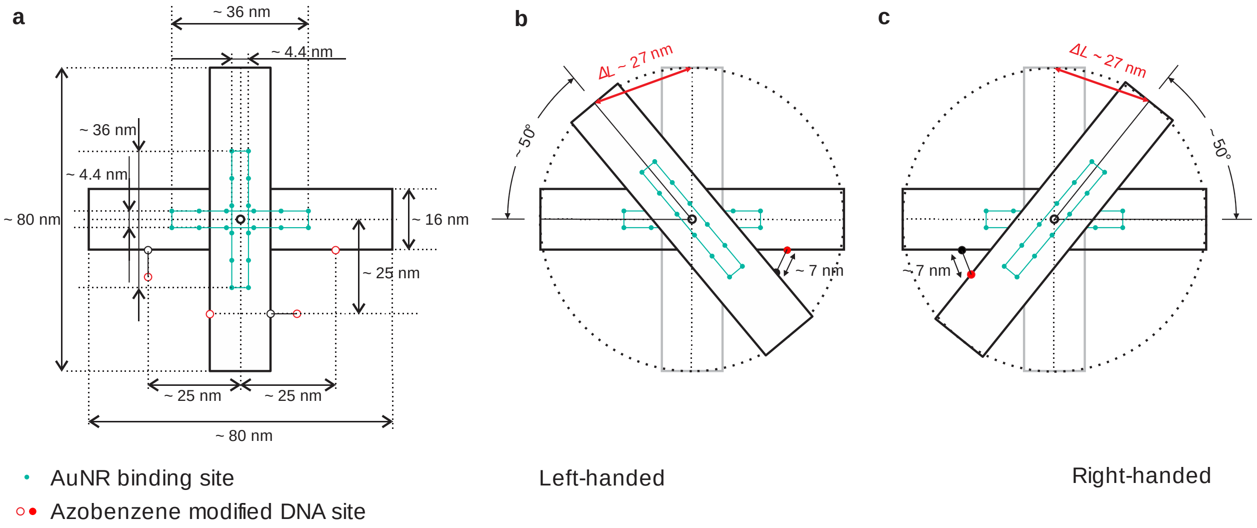

Supplementary Figure 2. Schematics of the DNA origami template.

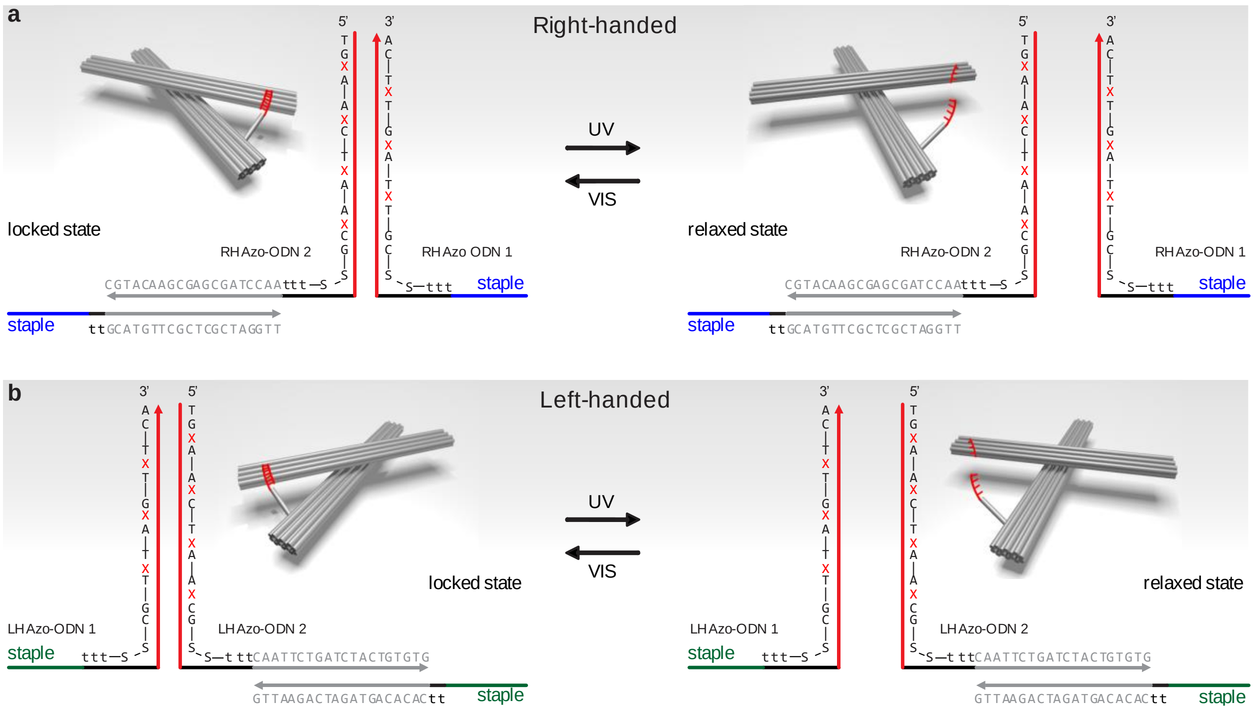

Supplementary Figure 3. DNA sequences used for switching of the DNA origami template. **a**, Switching between the relaxed and the right-handed states. **b**, Switching between the relaxed and the left-handed states. In the relaxed state, the two bundles of the DNA origami are not linked by the azo-modified strands. Hybridization of the Azo-modified DNA strands (Azo OND 1 and Azo ODN 2) results in the locking of the template into a configuration with well-defined handedness.

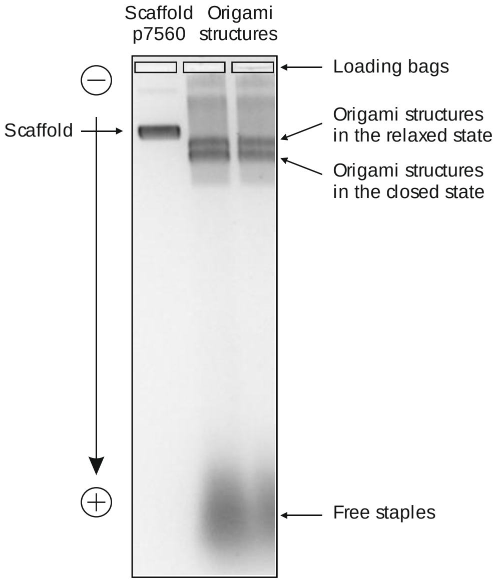

Supplementary Figure 4. **Purification of DNA origami by agarose gel electrophoresis.** Inverted photograph of a Sybr Gold stained 2.5% agarose gel under blue light (460 nm) illumination. The left lane contains the p7560 ssDNA scaffolds and the two right lanes contain the DNA origami structures after thermal annealing.

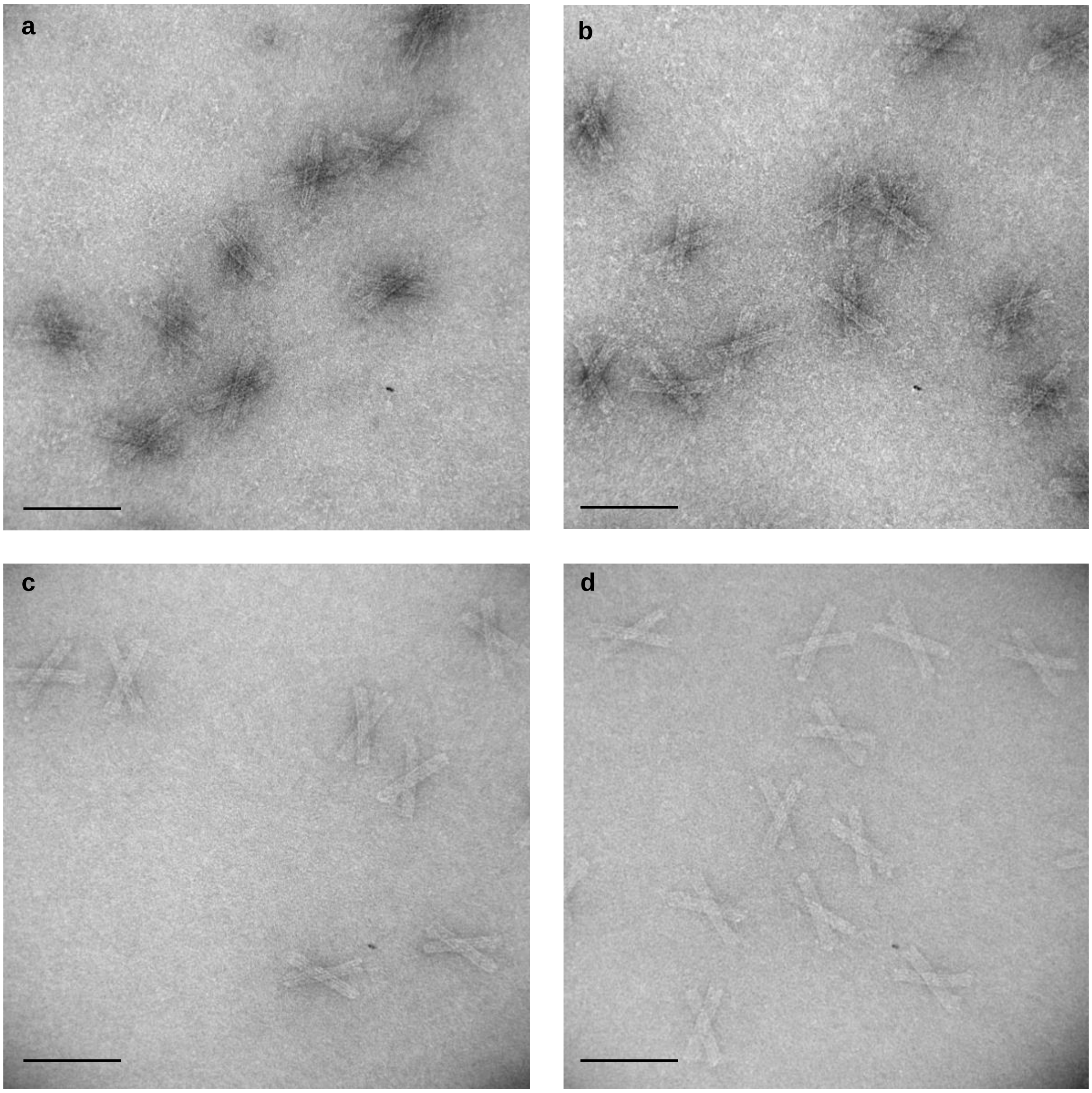

Supplementary Figure 5. **DNA origami templates in the closed state after the gel purification.** Right-handed structures (**a**, **b**) and left-handed structures (**c**,**d**). Scale bars: 100 nm.

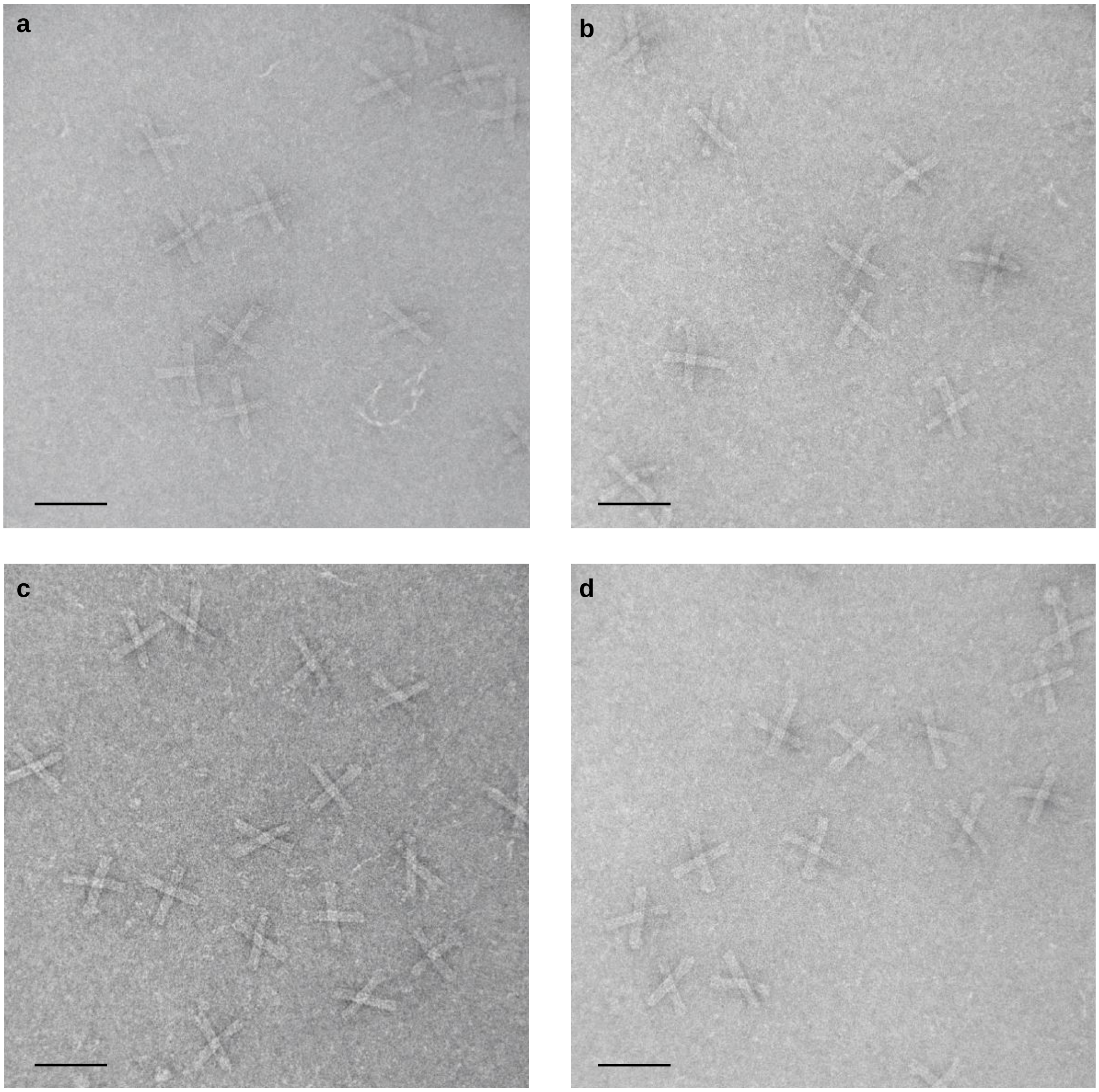

Supplementary Figure 6. **Right-handed DNA origami templates in the relaxed state after UV illumination.** Scale bars: 100 nm.

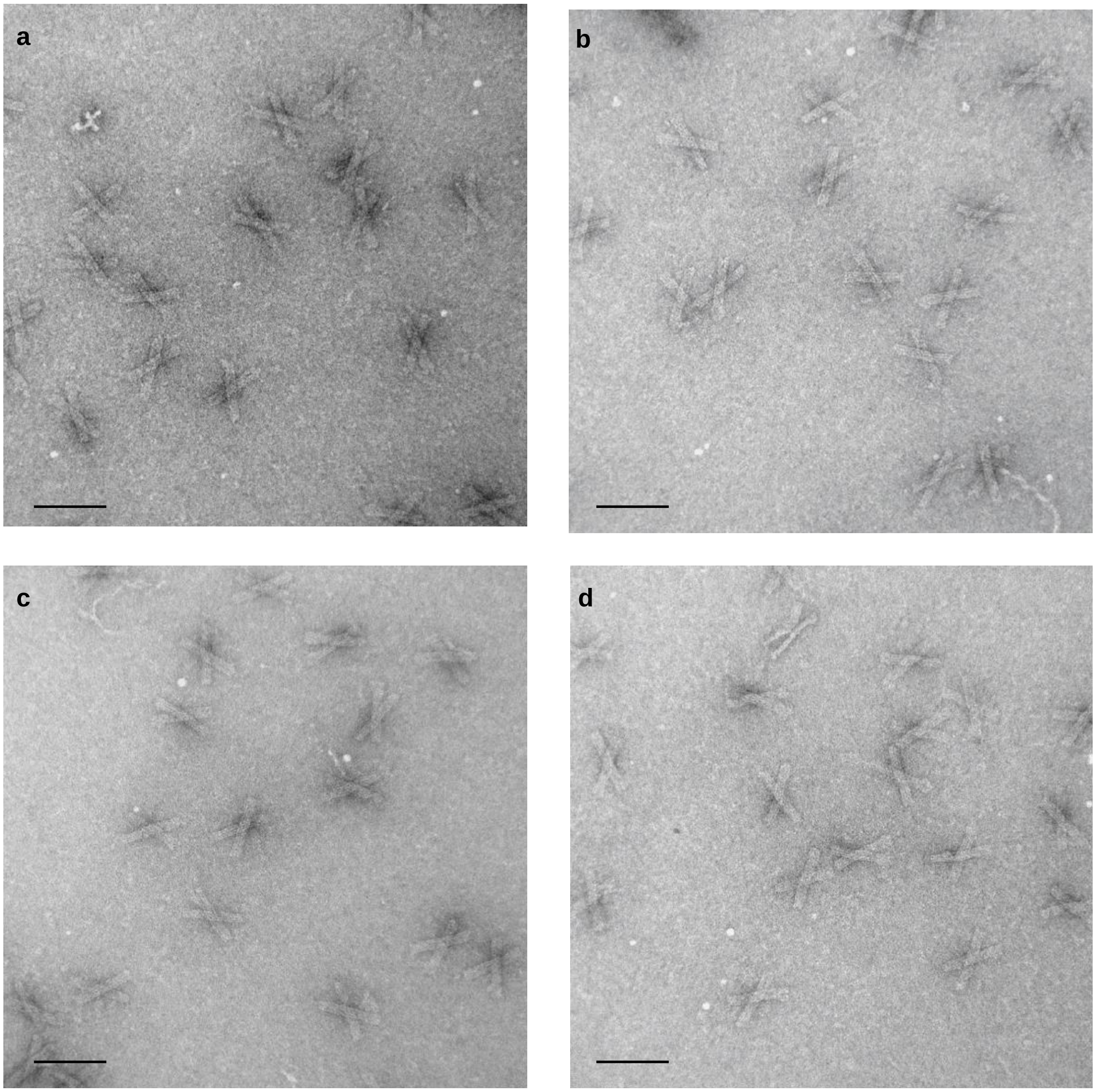

Supplementary Figure 7. **Right-handed DNA origami templates in the locked state after VIS illumination**. Scale bars: 100 nm.

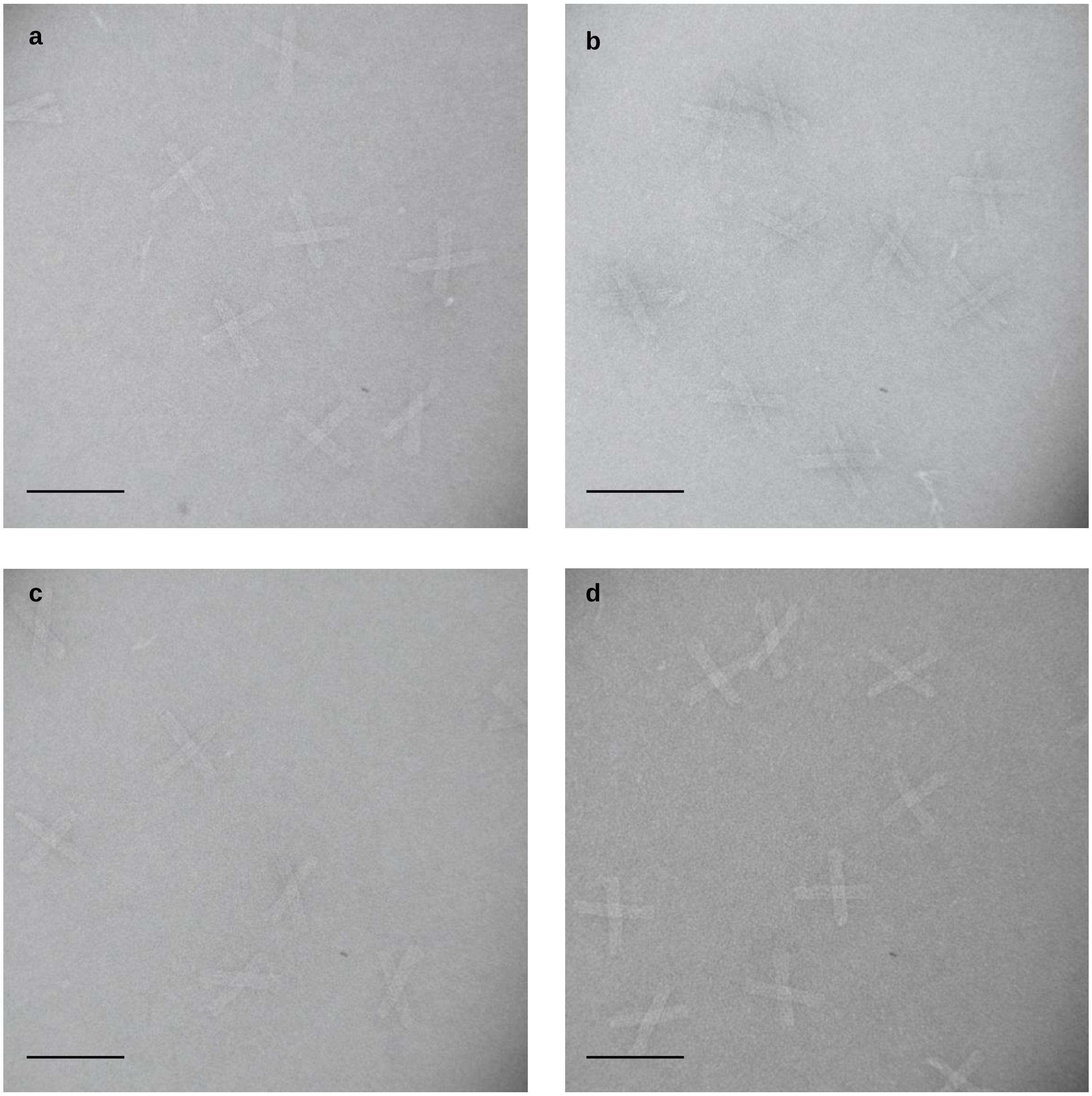

Supplementary Figure 8. Left-handed DNA origami templates in the relaxed state after UV illumination. Scale bars: 100 nm.

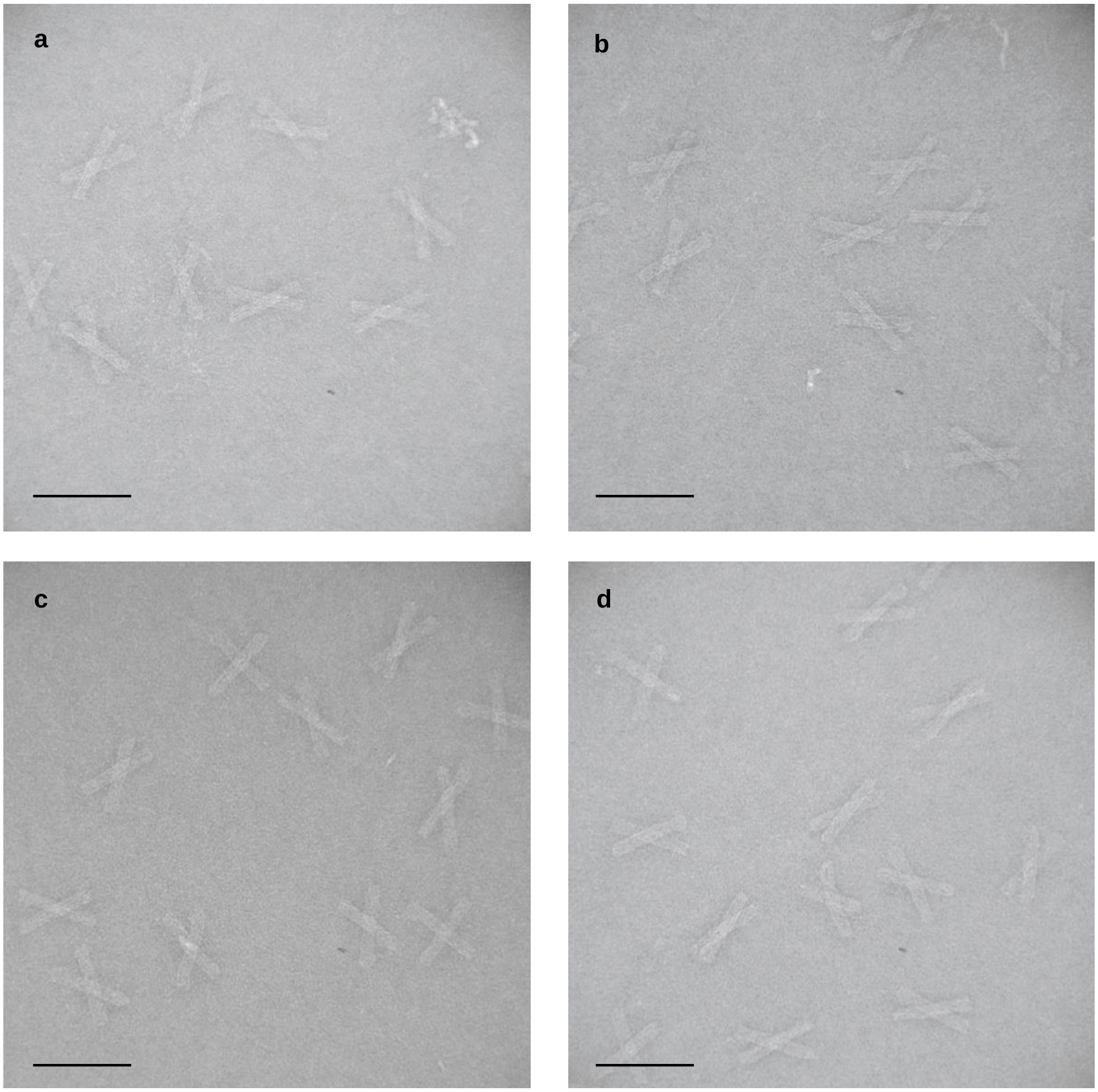

Supplementary Figure 9. Left-handed DNA origami templates in the locked state after VIS illumination. Scale bars: 100 nm.

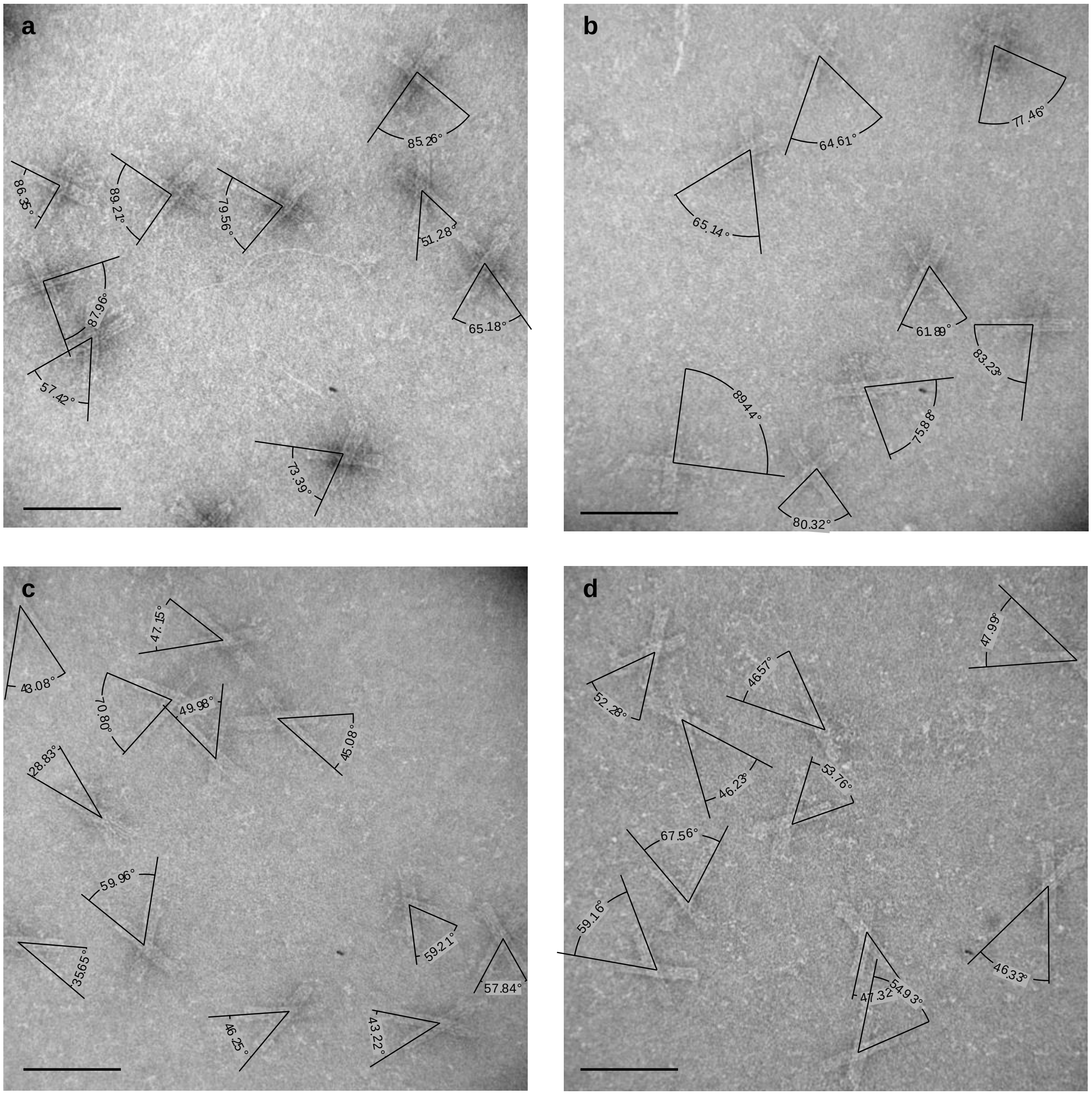

Supplementary Figure 10. **Examples of the acute angle characterization.** Right-handed structures after UV (**a**,**b**) and VIS light illumination (**c**,**d**). Scale bars: 100 nm.

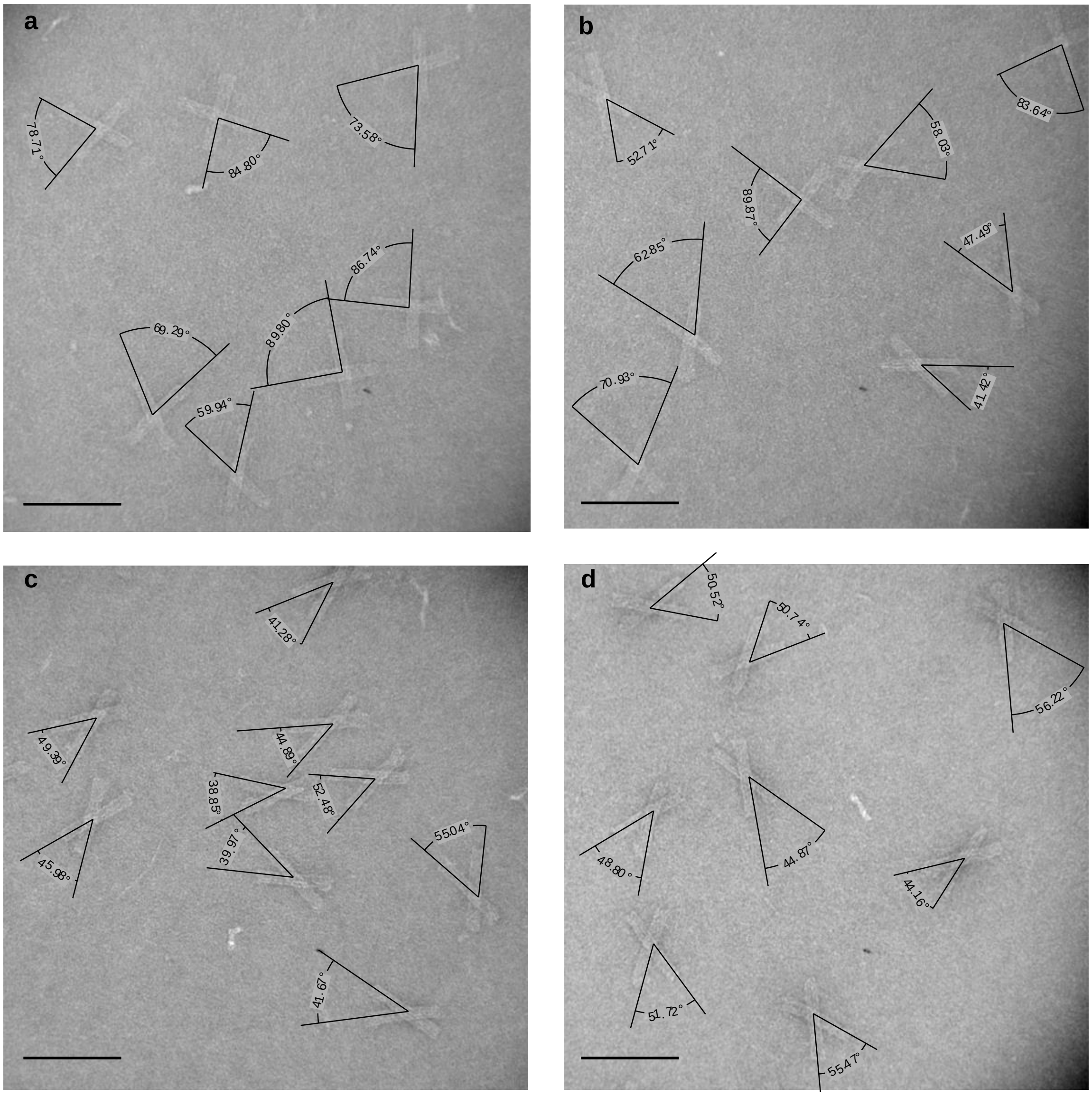

Supplementary Figure 11. **Examples of the acute angle characterization.** Left-handed structures after UV (**a**,**b**) and VIS light illumination (**c**,**d**). Scale bars: 100 nm.

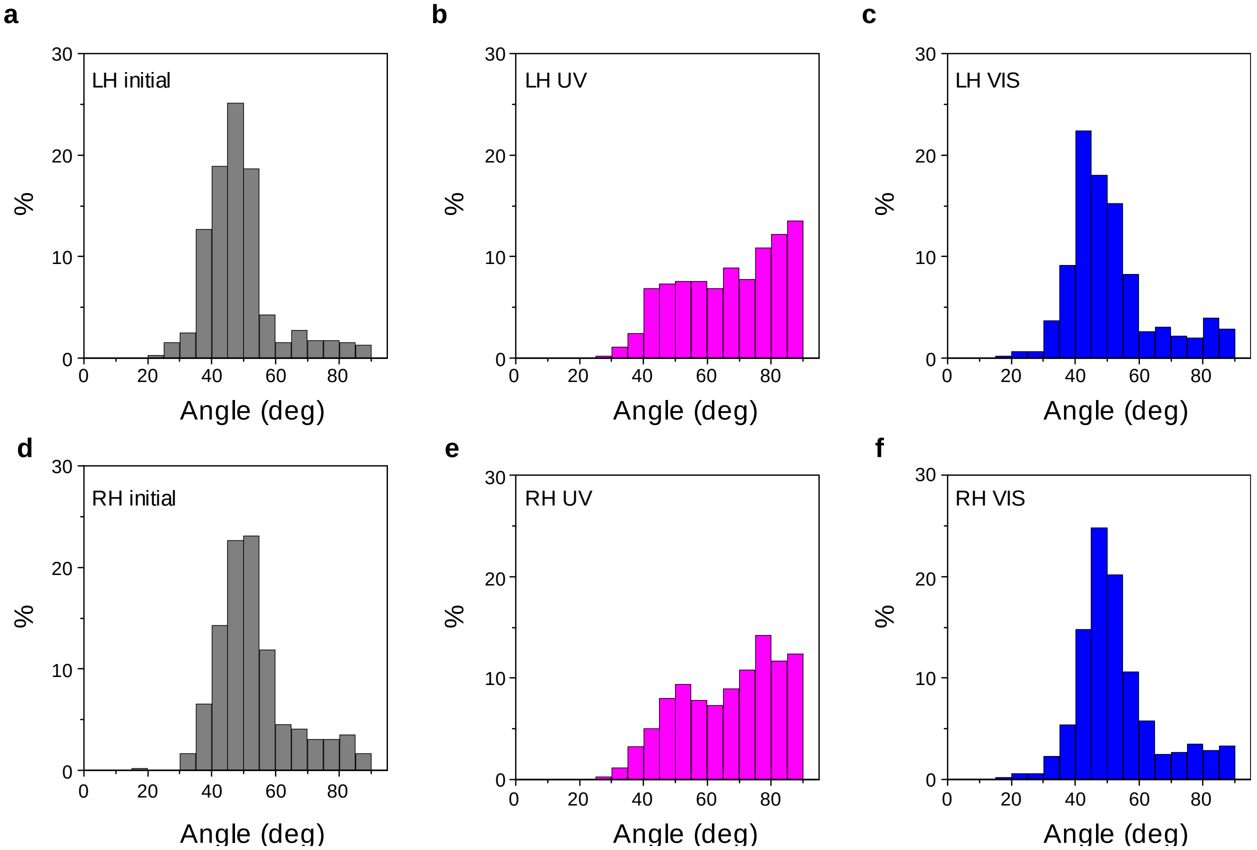

Supplementary Figure 12. **Acute angle distributions.** The left-handed DNA origami templates after assembly and purification (**a**), UV (**b**), and VIS light illumination (**c**). The right-handed DNA origami templates after assembly and purification (**d**), UV (**e**), and VIS light illumination (**f**). The number of analyzed structures: 402 (**a**), 452 (**b**), 460 (**c**), 510 (**d**), 463 (**e**), 541 (**f**).

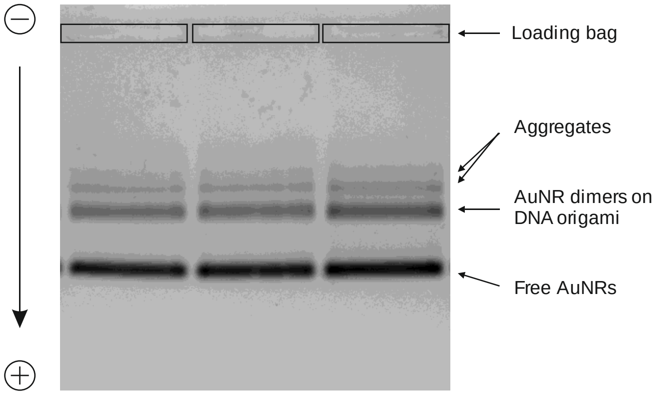

Supplementary Figure 13. Purification of plasmonics nanostructures. White light image of a 0.5% agarose gel. AuNR dimers are separated from the excess of AuNRs and AuNR-DNA origami aggregates. The lowest broad dark band represents the excess of the AuNRs, which run fastest, followed by the AuNR dimer band and the bands containing aggregates of the DNA origami template structures and AuNRs.

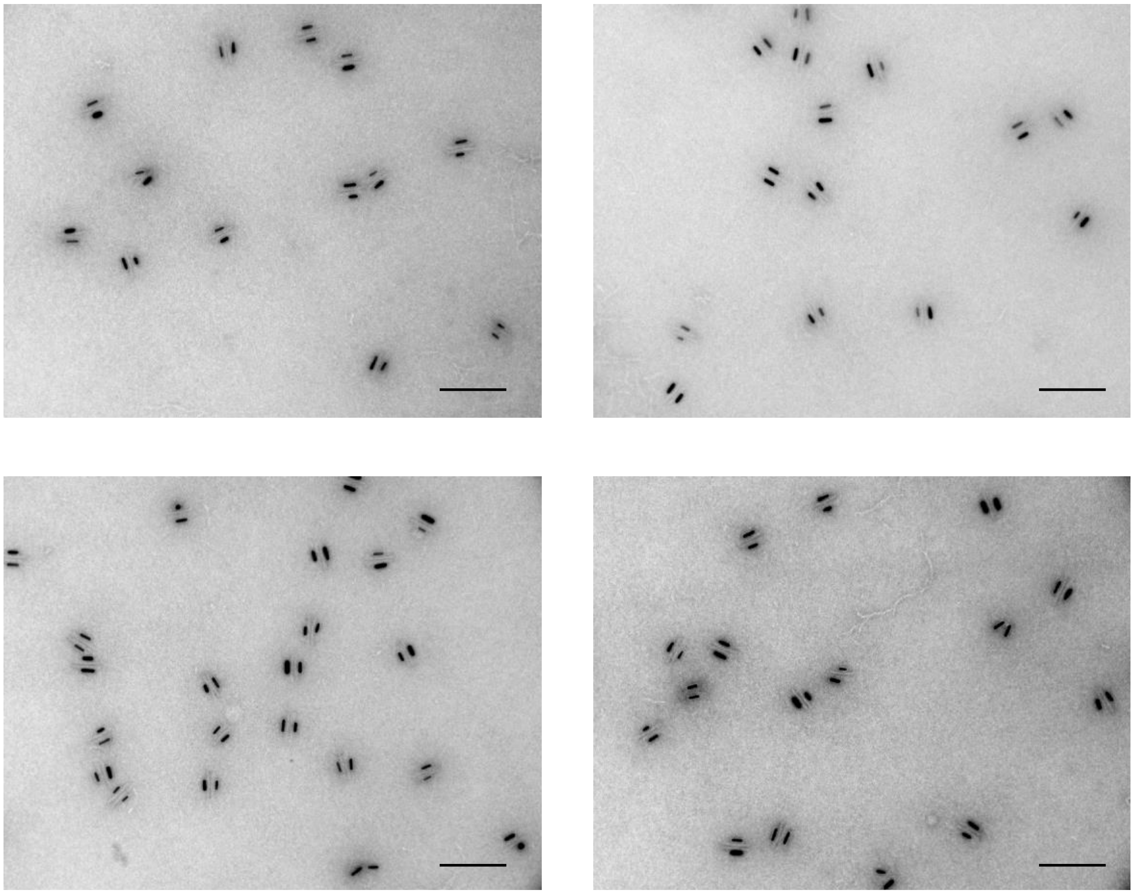

Supplementary Figure 14. Wide-field TEM images of the assembled AuNR dimers. Scale bars: 200nm.

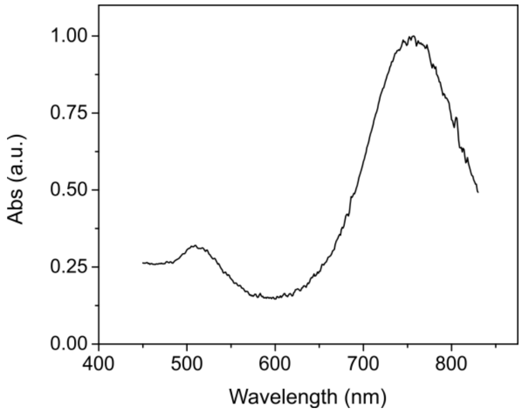

Supplementary Figure 15. Normalized UV-Vis absorption spectrum of the AuNRs functionalized with thiolated DNA after purification. The shorter wavelength resonance around 512 nm corresponds to the transverse plasmonic mode of the AuNRs, in which the plasmons are excited along the width of the AuNRs. The longer wavelength resonance around 755 nm corresponds to the longitudinal plasmonic mode of the AuNRs, in which the plasmons are excited along the length of the AuNRs.

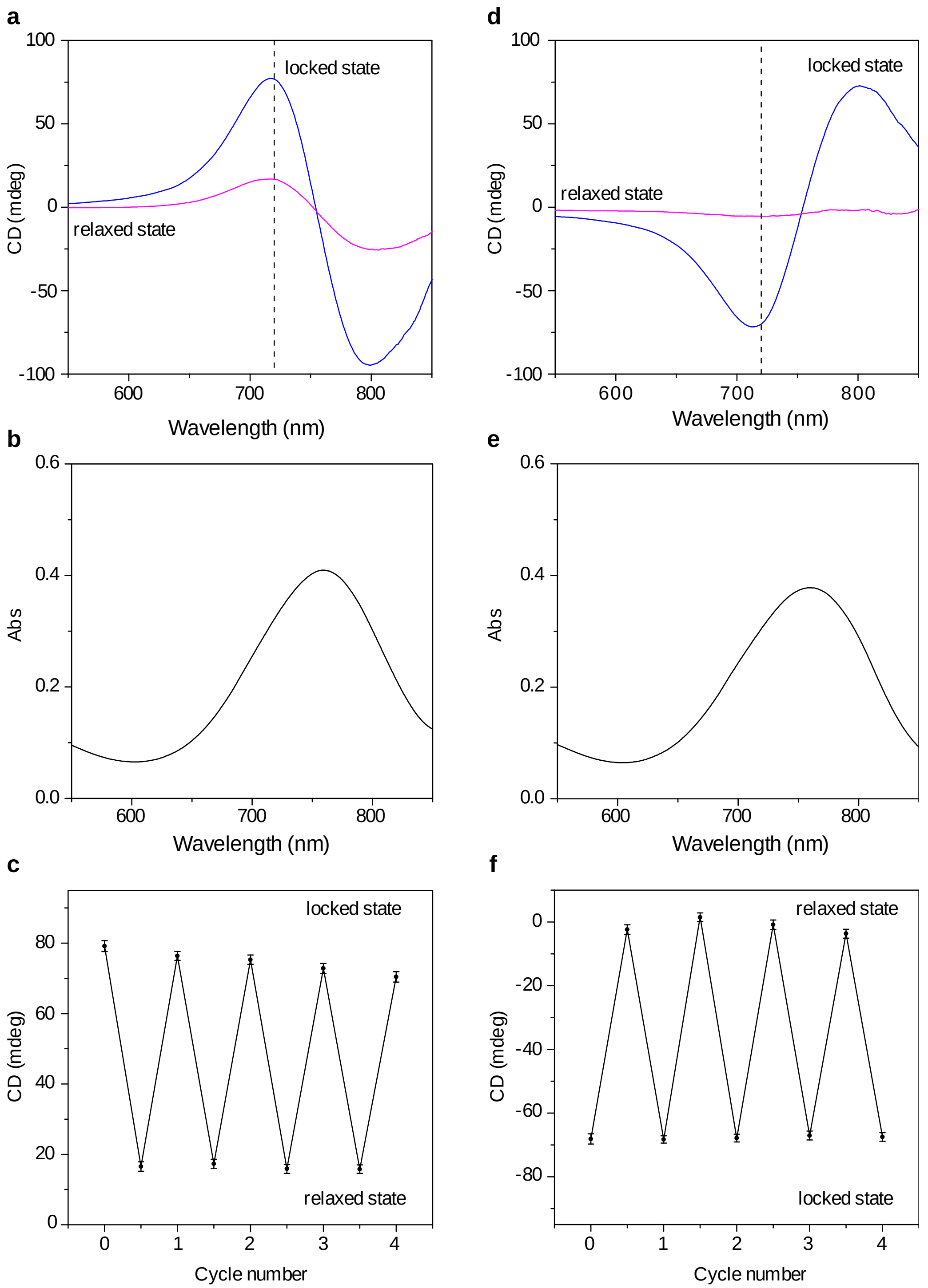

Supplementary Figure 16. **Optical characterizations of the light-driven left- and right-handed plasmonic structures.** **a**, CD spectra of the left-handed plasmonic structures. **b**, Absorption spectrum of the left-handed plasmonic structures in the relaxed state. **c**, CD intensity recorded at 720 nm (dashed line in **a**) during alternative UV and VIS light illumination in multiple cycles. **d**, CD spectra of the right-handed plasmonic structures. **e**, Absorption spectrum of the right-handed plasmonic structures in the relaxed state. **f**, CD intensity recorded at 720 nm (dashed line in **d**) during alternative UV and VIS light illumination in multiple cycles. The error bars represent one standard deviation from the mean.

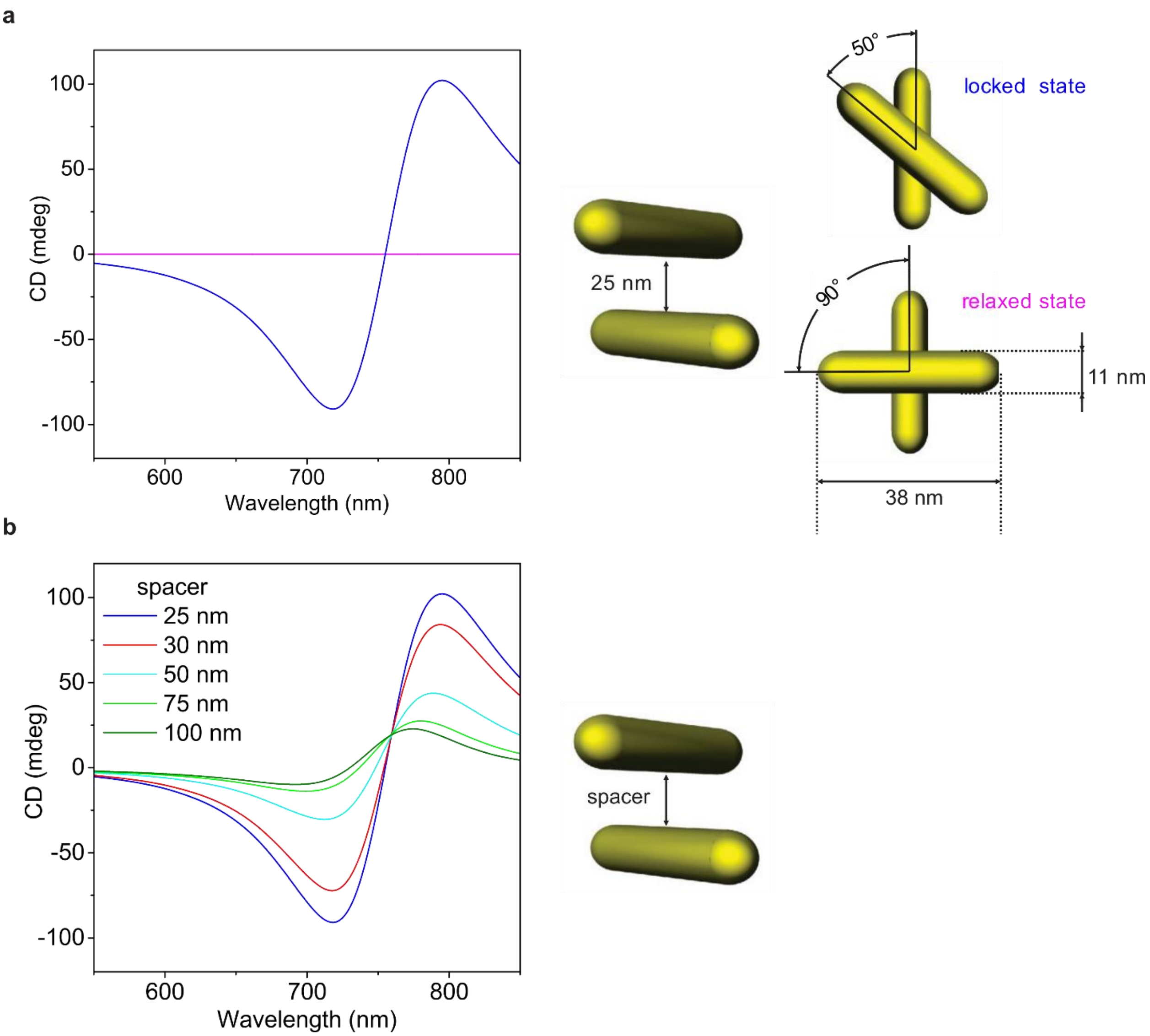

Supplementary Figure 17. Calculated CD spectra of the crossed AuNR dimers. a, CD spectra for the locked (right-handed) and the relaxed states. The calculations were carried out with the following parameters; AuNRs: 38 x 10 nm, surface to surface interparticle separation: 25 nm. **b**, CD dependence on the interparticle distance between the rods (in the right-handed configuration). The CD amplitude decreases as the interparticle distance increases.

Supplementary Table 1. Staple sequences of the DNA origami template.

Staples	Length (nt)		Start	End
AACGAGCGTCTTTCGCCATATTTGTTTAGAAGCGCATTAGAC	42	"core" staple	4[202]	7[202]
AGGTTTAACGTCGACACCGCCTGGAAGGGTTAGAAGATTTTC	42	"core" staple	22[62]	22[63]
TACTTTTTCTAATCTATTTACAGAAAGGTGCCTGAAACAGTGAGGCCAC	49	"core" staple	15[196]	25[202]
AAGAAGTCCAAAATTAGAGAGCTTAATT	28	"core" staple	4[118]	13[118]
TTTGAGATGTTTTTATAATCGCATTCGC	28	"core" staple	19[196]	23[195]
AATTTTAATTTTCAGCAAGGCAAAGAAT	28	"core" staple	26[146]	16[133]
TCGACAAATTATCAGTCAATATAGGTCTGAGAGACGTGAATTACGAATA	49	"core" staple	25[119]	14[112]
ACTCGTCAATCGGAGAGTAACCTCGTATGAGCACTGGAAGGTAACCTCA	49	"core" staple	14[132]	22[126]
CTCATAATGTTTAGACACCCCCAGCGATAAGAGGA	35	"core" staple	5[84]	9[90]
CATAACCTTATTACCCCTCAGAACCGCCCCAGGTCATTTTTG	42	"core" staple	2[97]	13[97]
CCGGCTTAGACGCTCGGCTGACGCATTTCCCAAATGAGCGGATGGAGGA	49	"core" staple	16[97]	25[90]
TGTCCATCACGCAAATTAACTTTTAGACAATAGGAACCCGTCGGCAAAT	49	"core" staple	25[217]	22[224]
AATTAATTTTCCCTCTCCTGGTTGGTGTGTGGACT	35	"core" staple	15[42]	27[48]
AGGGAGCGCCATCTAAAGCCTCAGAGCA	28	"core" staple	27[140]	15[153]
GTCAATACTAACTTTTTCAAATTAACACGCAAATGAAAAATC	42	"core" staple	24[104]	21[118]
TATTCTGAAATAAAGGTGGAATAAATTGAGGTTAAAGGTAGCAAG	45	"core" staple	0[261]	9[258]
TATACTTAGATACATTTCATCACAGAGGTCACCTTGCAGACA	42	"core" staple	18[139]	21[139]
GGCAATTTTCTGAATAATGGCAGTTACAAAAATTAATTACAT	42	"core" staple	25[63]	18[49]
GAGATTTACCTAAAACGAAAGGCCACTAAATCATTTTGGGAAGAATTCC	49	"core" staple	10[55]	2[49]
CCAAAGAAGAAAATACAAACAACTGGTAATAAGTTCAGTGCCTCAAGAG	49	"core" staple	11[224]	0[217]
GTGGTTGTGGGAAGGGAAACCAGGCAAA	28	"core" staple	15[252]	17[265]
CATACAGTTTGGGGAGTATCATAATGGTGAACCACTGAATGG	42	"core" staple	16[153]	21[153]
AACCGCCGAGGCAGGTCAGACGATTGACCACGCCACCCTCAG	42	"core" staple	5[224]	5[223]
GTTTGATGGTGGTTCCGTAGCCCGTGTTATCCTGTAAATCTCCCCGGGT	49	"core" staple	25[25]	16[24]
AACGGCTAGGGATAAAACTACAACGCCT	28	"core" staple	13[35]	1[48]
GGAGGTTACCCTCAACCACCGTAGCGCGTTTTCATATCGATAAGCACCA	49	"core" staple	4[237]	9[244]
CGAGTAAGTAATAAAGGGTGATATTTTAAATGCAA	35	"core" staple	25[203]	16[196]
AGAATCCAAATCAGTTGTAAACGTTAATCCCCAAAAACTAGCATGTCAA	49	"core" staple	24[216]	20[196]
ACCATCACACATAAGATAGCTTAGATTAAGGTTGG	35	"core" staple	27[77]	16[84]
GAGGTTTTTTTAGCGCGGGGTTTTGCTCAGAACTG	35	"core" staple	2[202]	13[195]
AATATCAAACCCTCTTGAAAGGAATTGAAACAACTTAGTTAATTTCGCA	49	"core" staple	22[125]	18[119]
GAAACACCAGAACGAGTAGTAGTGAGAA	28	"core" staple	4[62]	5[48]
AGCTAAACAGGAGGAATTCGCATTAAAT	28	"core" staple	24[265]	22[252]
AATTGCTGACGACGATAAAAATTTGCCAAGCGTCCACAGTTC	42	"core" staple	12[104]	6[105]
CTTGATATTCAACCTGAGAGTCTGGACCAAGGGATTTGACGA	42	"core" staple	18[244]	25[244]
AAAGAAATTACATCGGGAGACGCTGAGACATAAAACAGAAAT	42	"core" staple	22[83]	22[84]
AAGATTAGTATTCTGGCGGATAAGTGCCACTAATAACGGAAT	42	"core" staple	2[181]	13[181]
TTTGCCCGAACGCCCTGAAATGGATTTACTAAATTTATGCGT	42	"core" staple	25[140]	18[140]

AAGGATTCCTTATTGTCAGATAGCCGAACTGAATCCTTGCGG	42	"core" staple	0[216]	2[203]
CCTCATTTTGTCACAGAAAATACATACACATGAAAAACAGTT	42	"core" staple	3[238]	1[251]
GCATAGTATCAGTTTCAGGAGGTTTAGTCTTAGAGTACCTTT	42	"core" staple	2[118]	12[105]
ATTTAAACTCATTTTTTAACCAGGAACGCATTGCCGTTCTAG	42	"core" staple	22[223]	18[224]
CGTAATCAGTAGCGAGAGCCAGCAAAATTTGAGCCATTTGGGACCAGCG	49	"core" staple	8[230]	11[223]
ATTAACGAGGCGCAAACGGTGATCAAGA	28	"core" staple	8[90]	6[77]
GAGGCTGCAAACGTAATCAATCAAAAGGCACCGACCACCAGTGCAGCAC	49	"core" staple	0[237]	8[231]
AAACAGGACGTCAAAAATGAATTACAAATAGCCATATTTAACCGGAGCG	49	"core" staple	7[182]	9[174]
AATACGTGGCACTGTATCTAAAAATTTACATTGGCGCGTAAG	42	"core" staple	22[146]	22[147]
TTAACGATTGCCTTGATATTCTCATATGACGCAGT	35	"core" staple	2[216]	13[223]
TTCGAGGAATTGTAAGGAATTGCGAATAACAGTTTACCATCGGCTTGCA	49	"core" staple	8[41]	11[34]
AAGACGGAGGAGTAAACAGGGCTTAAGCTA	30	"core" staple	15[22]	14[18]
GAGGAAACGCAAGGTAATTGAGAATCGCTATCTTAGGAAACC	42	"core" staple	12[181]	12[182]
ACAGACATTAATTTCAACTTTCGAAGGCCTTTGAG	35	"core" staple	2[48]	13[55]
ATGTTAGAGACTCCTTGAGTAACAGTGCGGAGTGTAATAAAT	42	"core" staple	13[224]	3[237]
CAATATCCTATGGTGTGTAGCGGTCACGGTGCTGACCTCTAA	42	"core" staple	17[238]	15[251]
AATAAATTCGAGCTCAGGTCAGGACGAGTAGATTTAGCCAGT	42	"core" staple	10[125]	10[126]
CGGAGACTACTTCTCGGGCGCTAGGGCGGACAATGACGCAAGATGTG	47	"core" staple	17[217]	36[226]
TATTACCGCCTTGCTGAAAAGAACATCC	28	"core" staple	25[161]	16[161]
TTTGGGGCTTGAATGAGAAGAGTCAATATACCTTTTTAACCT	42	"core" staple	27[98]	16[98]
CATTTATATTATTTGCACGGTTGGCAAA	28	"core" staple	24[90]	23[104]
ACTAATCGGTTTATTGACCAACTTTGAACCGGATAAGGAACA	42	"core" staple	6[46]	6[47]
TGATTGCAGTAACAGTACCTTTTGCGTACCTACCA	35	"core" staple	20[69]	23[76]
GCACGTAGCGCGTATGGTGCCGGCGATCGGTGCGGGCCTCTATCACCAT	49	"core" staple	25[245]	17[237]
ACCAGAACCGAAGCCAATGAAATAACCCACCCTGAACAAAGT	42	"core" staple	12[195]	8[182]
GGGAACAAGTAACAACGCCATCAAAAATCCGATTAAGTTTGAGGCACCG	49	"core" staple	21[238]	18[245]
ACCCAAAAGTACCAAAGAACGCGAGGCGTGAAGCCGCTACAA	42	"core" staple	13[182]	3[195]
TTATCAATCCCATCCTCATTGAATCCCCAGGTCTTTACCTGT	42	"core" staple	7[140]	7[139]
AGTAATAAAAGGGACTCATGGGCGTTAAATAAGTT	35	"core" staple	23[175]	19[195]
TTAACCTGCCTAATGAGTGCACCTGAGACAAAATCCCATGAT	42	"core" staple	18[48]	25[62]
CTATTAGCCTGAAAAGATTCACCAGTCATGACGCTACCGACCGAAAAAG	49	"core" staple	21[154]	18[161]
AGAGCCAAATCTACGTTAATACAGGACGGTGAATTCTGACGA	42	"core" staple	0[62]	4[63]
GGGAGTTAAAGGCAGTATCATCGCCTCACAGCGGAAATTGGGCTCTGTA	49	"core" staple	11[35]	4[28]
GTTATTACATAAATACATAGCATCATTTCTCCGAAGGCCCACCCTGATT	49	"core" staple	16[83]	26[63]
CTTAAGTGTCCTTACTGCGCGCTACAGGTAACGTG	35	"core" staple	14[265]	25[258]
TTTGCGGAGATGGTGCCCTCATAGTTAGCCAGTACGCAAGCCCAATAGG	49	"core" staple	12[41]	0[21]
GTGTGATTCGCCATTGCGCGAACTGATAACAGAGATAGAACC	42	"core" staple	19[168]	22[168]
AATGCCCTACATGGGAATGGAGAGCCGC	28	"core" staple	1[252]	4[252]
AGCACTAGGTGGGCTATCAAAATAAATTAAGCAATGTAAGCA	42	"core" staple	27[119]	14[133]
CAGAGGGTAATTACAATAAACTGTACAGAGAGAATAACATAA	42	"core" staple	8[181]	7[181]
CATTTGAATAAATCCCAACGTGGGCGATCTCTGACTAGAATC	42	"core" staple	17[49]	15[62]
AATAATACAATAGATAAGTCCAACATGTTTCTGTC	35	"core" staple	6[153]	9[160]

GCTGAATACCGTACGAGATTTAGGAATAAAAGGAAGGGCACT	42	"core" staple	13[119]	3[132]
TATCAAATGTTTGGCGAATTATTCATTTCCAAACAATAACGG	42	"core" staple	23[77]	20[77]
GGGAGAGTAAGAAACGATTTTTATTTATCAAGAAACCTTTTT	42	"core" staple	7[203]	11[202]
ATTAAATTTTTGTTTGAGAAGGATCTACCCGGAGAGGGTAGCATTCAAA	49	"core" staple	23[196]	17[202]
GTAGCAACCACCCTCATTTTCACAGAGGACCCGCT	35	"core" staple	1[49]	12[42]
TGAAACCCGGCATTCCGGAACCAGAGCCGAGCCACTCACCGTGCGACAT	49	"core" staple	8[251]	11[244]
AACGCCACCACATTGGAATAGGTGTATC	28	"core" staple	2[139]	0[126]
TAGAGCCTCAACAGAATCAATATCTGGTGCCAGCACGCCTGC	42	"core" staple	24[111]	20[98]
AGAAAAGGCTGGCTGACCTTCTACAGACCAGGCGCCAAAAAG	42	"core" staple	6[104]	8[91]
ATCATTCTTTTATTAAGTTTCATTCCATCCAACGCTCAACAG	42	"core" staple	4[160]	11[160]
GCTTGCCACCTTATGTAAAATTCATGAG	28	"core" staple	4[76]	13[76]
TAATAGTTTTACCACCTTTTGATAAGATCACTCATCTTTGCGGAAGCAA	49	"core" staple	4[97]	11[104]
CAGACGAAACGCCAATCAATAATCGGAGAATAGCAGCCTTAACAAGAAA	49	"core" staple	9[161]	6[154]
ATCAGTTATTACCTTGTGAGTGAATAACCTTGCTTCGAATTT	42	"core" staple	26[62]	17[48]
TAGAATTCATTACCCAAATCATGACAAGAAAGAGG	35	"core" staple	5[49]	7[69]
CAGATATGGGTTGATATAAGTTTTTAAATATGCAA	35	"core" staple	1[154]	13[160]
ACTCACAACGCGCGGGAGATGAATATACTTTGAATACAGCTA	42	"core" staple	20[48]	20[49]
CAAAAGGAGCCTTTTGAATTTCAATGACAAGATAAATTGTGTGCTTGCT	49	"core" staple	7[21]	8[42]
TCAACCGGTTTATTAAAGCCACTTTTGATGATACACCGTATAGTATTAA	49	"core" staple	11[245]	0[238]
CATCGAGTTGATTCTGTAGCTCAACATGATAGCCCCAACTAATGCAAAT	49	"core" staple	3[133]	1[153]
TTTTATCCAAAGTTGCATGATTAAGACTAGGATTAGAACCTCCCGGGGT	49	"core" staple	3[196]	1[216]
AGAAAAGATTTTGTTAAAATTCTGGCCAGCCGGTTGATAATC	42	"core" staple	21[196]	21[195]
AAAGTAATCAGCTAATGCAGAACGCGCCTACCGACAAAAGGT	42	"core" staple	9[147]	9[146]
AAAAGAAAAATCGGGAGTTGCTGCCCTTGAGGCGGTTTGCGTTCGGC	47	"core" staple	26[48]	21[39]
AGCTGATAGCAAGCCTGGGGTCACAATTCCACACAACAT	39	"core" staple	23[35]	18[24]
ACTCCAATCAAAGCCAAATATAAAGCGGATTGCATATACGAGAATGACC	49	"core" staple	11[105]	7[111]
ATAAATCCAGAAGCCGCGTTTTAGAGAATATAAAGCTGACTA	42	"core" staple	7[112]	8[126]
GCTTTCCGGGGACGCGTAACCGATTGACCGTAATG	35	"core" staple	18[258]	21[265]
ACAGATGGACGGTCTGTTACTTAGCCGGTATACCAAAAGCTG	42	"core" staple	7[70]	5[83]
AAGAAAAGTAAGTTAACAATAATAAGAGCCCAATCCAACGCT	42	"core" staple	11[203]	4[203]
CAGACTGGAACCGCCTCCCTCAGAGCAATTTGCCTTTAGCGT	42	"core" staple	7[224]	7[223]
AGAGCCGAAAATCATTCGGTCATAGCCC	28	"core" staple	5[252]	7[265]
TTACCATTGAATTACACCCTCCGCCAGCATTGACA	35	"core" staple	9[245]	4[238]
AAACCAAGTACCCTGTCATAAATATTAA	28	"core" staple	4[139]	5[139]
ATAATCCATTATCAAGATAACTATATGTAAGATGA	35	"core" staple	25[77]	18[84]
GCTGCATTAATGAAATTGGGCGGGCAAC	28	"core" staple	21[21]	23[34]
TCGGAACGAACCCTCACGCTGAGCCCACGCATA	33	"core" staple	13[19]	10[18]
TACCAGTAAACGAAGAACCGCCACCCTC	28	"core" staple	2[76]	0[63]
TAAAGCATGAGGCGGTCAGTATATATTTAATAGAT	35	"core" staple	21[119]	24[112]
GTGAAATAGATAGGCACTATTAAAGAACAATGAGTCGCTATT	42	"core" staple	17[28]	15[41]
ATTGTCACCAGGGTTTTCCCATGTAAAACCAGG	33	"core" staple	14[244]	37[249]
GTGAGCGAACGGCGGTGCATCTGGCAAACAAGAGATCTCCGT	42	"core" staple	22[251]	21[237]

GTAATCTACGTAACAGCGCGAAGAATACACTAAAATAAACGG	42	"core" staple	6[76]	12[77]
CTTGAAACAATATATTTTTAAAGAAAACAAATCGC	35	"core" staple	15[63]	19[69]
CCCAATAGCAAGCAAAGCCGTCAAGAACGCATGTAGAAACCAACATGTA	49	"core" staple	2[160]	10[154]
CATTAATTGCGTTGCGGTAAAGCGGTCCACGCTGGTT	37	"core" staple	21[40]	24[21]
AATAAATTAAAGCTGACAGTGCGGCCCTCCCCGATATTTATT	42	"core" staple	16[160]	26[147]
AGATTGTATAAGATATCGATGAACGGTAATCAGGTGTACGCC	42	"core" staple	21[217]	24[217]
CGTGGCGGCTCGCCTTATGACCCTGTAATGCCTGA	35	"core" staple	27[182]	16[189]
CAGGAAAGTAGAAGACGTGTAGGTAAAGTAAATAA	35	"core" staple	25[182]	18[189]
GTAGCAAAGTCAAAAAAATTTTTAGAACCCTCATAGAAAGGC	42	"core" staple	26[230]	17[216]
GACTCTAGGAAAGCACTATCGGCCAGCCTACATTTCACGACC	42	"core" staple	14[174]	23[174]
TTGACGGTGATACCAAATCGGTTGTACCTAGCATTGTGGCATATTACTA	49	"core" staple	27[161]	18[168]
TCTAGCTGGCGAGGAGAAGCGACGGC	26	"core" staple	36[249]	38[238]
TAAATGAATTTTTGGATCGTCGGGTAGC	28	"core" staple	3[21]	13[34]
GTAATTAATAGTAGAAAAACACTGGAGT	28	"core" staple	16[188]	14[175]
CGAGCTTTCTCAAAGGGGGGATTCGCTATTACG	33	"core" staple	37[226]	15[244]
CAGTGCCGGTAACGACCTTATCTGGCAATGCCGTT	35	"core" staple	38[237]	26[231]
**Right-handed structures only**				
AAGCCTTTATTTCATCCCGCCAAAATAAAAAGGAGTTGATTAAAGAGTC	49	"core" staple	15[210]	25[216]
**Left-handed structures only**				
AGGGAACCGAACCACGAAATCCGCGACCTACAACG	35	"core" staple	8[62]	10[56]
**Modified staples and Azo modified oligos[1]**	**Length (nt)**		**Start**	**End**
CGGATGGACCGCCAAGGTAGAAAGATTCAAGAGCAACACTAT **AAAAAAAAAA**	52	nanorod handle	13[98]	2[98]
AACAGTGCAATTACGATAATATTTAGAA **AAAAAAAAAA**	38	nanorod handle	20[97]	25[97]
CTAATATCAGAGAGATAGCAAATAAACACAGAGCC **AAAAAAAAAA**	45	nanorod handle	9[175]	4[182]
AAGACAAAGAACGCGAGAAGAGCAAAAGAAATGCTCCAGAAGCAAGTTT **AAAAAAAAAA**	59	nanorod handle	18[118]	27[97]
TAGGGCTTGTCTGGTTCATCGTAGGATGCCCTGTCTTTCCTT **AAAAAAAAAA**	52	nanorod handle	11[161]	4[161]
GTATTAGGAAACCAGATGCAAATCCAATCGCAATGTTTTGCGGCCGTAA **AAAAAAAAAA**	59	nanorod handle	25[98]	27[118]
GAAGTTTACCCTCACTAACGGAACAACACTACTGGCTCATTA **AAAAAAAAAA**	52	nanorod handle	13[77]	2[77]
CTAAAGTGTCGAGAAGAAGGCTTATCCGGTATCATTACCGCG **AAAAAAAAAA**	52	nanorod handle	13[161]	2[161]
TGAAACAAACATCATGGAAACTCATATTTACGTGA **AAAAAAAAAA**	45	nanorod handle	18[83]	27[76]
AGCCCGAAAGACTTGAACCAGACTGGATGAGGGGG **AAAAAAAAAA**	45	nanorod handle	9[91]	4[98]
CCTGTTTCGCGAGCTGGTAATTTAGAGC **AAAAAAAAAA**	38	nanorod handle	18[160]	27[160]
TTATAGTAAAAATCCTCAAATGCTTTAAAATACTGCGGAATCTTTGCAA **AAAAAAAAAA**	59	nanorod handle	8[125]	4[119]
ATTCGCCGCAGAGGATTATACCATCAAT **AAAAAAAAAA**	38	nanorod handle	20[76]	25[76]
GCGATTTTAAGACGAATCAGTGAATAAG **AAAAAAAAAA**	38	nanorod handle	3[77]	4[77]
TCATATGTACCCCCTAAAACAAAATAAGAAATACCATTGCAA **AAAAAAAAAA**	52	nanorod handle	20[195]	25[181]
TAATTTGCTATTTTGCACCCATTAAATC **AAAAAAAAAA**	38	nanorod handle	4[181]	2[182]
TAGCACAACCTGTTTAGCTATAAAGTTTACCCTAA **AAAAAAAAAA**	45	nanorod handle	16[132]	27[139]

CTTCTGATCTTTAATAAAAATACCGAACTTGAAATCAATCGTAGAACAA **AAAAAAAAAA**	59	nanorod handle	22[167]	25[160]
ACACCGGAATCATACAATTCTAACTCAACGGCGAA **AAAAAAAAAA**	45	nanorod handle	18[188]	27[181]
ATTTTCGAGACCAGTATAAAGATAACAGAACAAGCGATACAT **AAAAAAAAAA**	52	nanorod handle	10[139]	2[140]
ATTTAGGCAGAGGCTTTACGAGGGTATT **AAAAAAAAAA**	38	nanorod handle	10[153]	4[140]
TAGGGGCTCGAGGTGAACAAAACTTTACAAACAAT **AAAAAAAAAA**	45	nanorod handle	14[111]	25[118]
ATAATGCCCAATTCTGCGAATAGCGAGATTACGAG **AAAAAAAAAA**	45	nanorod handle	13[126]	2[119]
ATATTTTCAGCAGAAGATAAATTCTGACTCTTTAGTAAATCC **AAAAAAAAAA**	52	nanorod handle	21[140]	25[139]
**Right handed**				
GACTAAAGACTTTTACGTAATAGGCAAAAACAAAGTGCTCCAAATCATA– **–tt GCATGTTCGCTCGCTAGGTT**	71		13[56]	8[63]
CTGATAAATTAATGAAAGGCTATCGTAAAACAGGA– **–ttt–S–S–CGTXTAXGTXTCA**		RH Azo-ODN 1	18[223]	21[216]
TGXAAXCTXAAXCG –S–S–ttt AAC CTA GCG AGC GAA CAT GC		RH Azo-ODN 2		
**Left handed**				
CATCACTAAGGGAAGAAAGCGCCCCGCTGCGGGAG– **–tt CACACAGTAGATCAGAATTG**	57		26[202]	15[209]
CAAATTAACTGAACACAAGAATTGAGTTAAGCCTTACAGAAT– **–ttt–S–S–CGTXTAXGTXTCA**		LH Azo-ODN 1	8[209]	8[210]
TGXAAXCTXAAXCG –S–S–ttt CAA TTC TGA TCT ACT GTG TG		LH Azo- ODN 2		

[1] Modifications introduced to the staple strands are highlighted with bold fonts.
Thymines used as spacers are highlighted with the lowercase font, t.

Supplementary Table 2. Thermal annealing temperatures and times for DNA origami folding.

**Temperature, °C**	**Time, min**
80	15
79, 78, …, 72, 71	1
70, 69, 68	5
67, 66, 65, 64, 63	10
62	15
61	20
60	30
59, 58,…, 39, 38	60
37, 36	45
35	30
34	20
33, 32	10
31, 30,…, 26, 25	5
24, 23, 22, 21	2
20	Hold

Supplementary Methods

Design of the DNA origami templates

The DNA origami template structures were designed using caDNAno software[1]. The origami template consists of two 14-helix bundles (80 nm × 16 nm × 8 nm) linked together by the scaffold crossing over twice between them at one position (Supplementary Fig. 1). The two crossings have an 8 nt region of the unfolded scaffold, which serves as a spacer. Twelve binding sites along two parallel lines are extended from each origami bundle for robust assembly of one AuNR (see Supplementary Fig. 2). The length of the two lines (~36 nm) is designed to match the length of the AuNRs (38 nm). The distance between the two lines is ~4.4 nm.

The DNA origami template structures were functionalized with additional ssDNA strands with azobenzene modifications for controlled light-induced conformation switching. Specifically, the structures can be switched between the relaxed and left-handed configurations, or the relaxed and right-handed configurations.

Azobenzene-modified DNA strands

Four DNA strands modified with azobenzene modification were used (RH Azo-ODN 1, RH Azo-ODN 2 for the right-handed and LH Azo-ODN 1, LH Azo-ODN 2 for the left-handed configuration, see Supplementary Table 1 and Supplementary Fig. 3). These four strands were obtained from two azobenzene-modified segments (Azo-ODN 1 and Azo-ODN 2) linked to four different single-stranded DNA strands through disulfide following the previously described procedure[2,3]. RH Azo-ODN 1 and LH Azo-ODN 1 were linked to the staple strands, whereas RH Azo-ODN 2 and LH Azo-ODN 2 were linked to the DNA strands which constitute part of the double-stranded branch linking the two DNA origami bundles together into the locked configuration (see Supplementary Fig. 3).

Azo-ODN 1 and Azo-ODN 2 contain three and four azobenzene modifications, respectively. Multiple azobenzene modifications are required for efficient photoregulation of DNA hybridization[4,5].

Folding of the DNA origami template structures

For preparing the DNA origami template structures, the sample prepared for thermal annealing contained: 10 nM of the p7650 scaffold, 100 nM of each unmodified staple, 30 nM of the modified staples 13[56]-8[63]/26[202]-15[209] for the right-/left-handed structures, and 50 nM of Azo-ODN 1, Azo-ODN 2 modified staples and oligos. All DNA strands were mixed in 1x TE buffer (10 mM Tris, 1 mM EDTA, pH 8) together with 20 mM of $MgCl_2$ and 5 mM of NaCl. The mixture was then exposed to a thermal annealing ramp; first heated up to 80°C for 15 min, then slowly cooled down to 20°C (see Supplementary Table 2).

Purification of DNA origami structures

The DNA origami template structures were purified by gel electrophoresis (Supplementary Fig. 4). After annealing, the sample contained DNA origami structures in both “relaxed” and “closed” states. To purify structures in “closed” state from those in “relaxed” and extra free staples, we ran a 2.5% agarose gel in a 0.5 x TBE buffer with 11 mM $MgCl_2$. After staining with Sybr Gold (Invitrogen), favored band (labelled as “origami structures in “closed state” in Supplementary Fig. 4) was cut out and the DNA origami template structures were extracted with Freeze `N Squeeze spin columns (BioRad).

The concentration of the origami structures after purification was estimated using UV-Vis absorption spectroscopy. For estimation, we used an extinction coefficient of 1.3 $M^{-1}cm^{-1}$ at 260 nm. The purified templates in “closed” state were later used for AuNR assembly. TEM images of the purified DNA origami template structures are shown in Supplementary Fig. 5.

Functionalization of the AuNRs with DNA

Functionalization of the AuNRs with thiolated DNA (SH-5′ $T_{16,}$ biomers.net) was carried out following the low pH route[6,7]. One milliliter of 1 nM AuNRs was mixed with 50µL of 1% sodium dodecyl sulfate (SDS), 100 µL of 10X TBE, and 5 µL of 1mM DNA. Hydrochloric acid (HCl) was used to adjust the pH value of the solution to 3. Before being added to the AuNR solution, thiolated DNA strands were incubated with Tris(carboxyethyl) phosphine hydrochloride (TCEP) for at least 30 min in order to reduce the disulfide bonds.

After incubation of the AuNRs with DNA for 1 hour, 0.5 M NaCl was added to the solution. The solution was then gently shaken for at least 3 hours. During this step, the solution became slightly cloudy due to the fact that cetrimonium bromide (CTAB) was replaced from the surface of the AuNRs by thiolated DNA. Upon being warmed up to ~ 35°C for 5 min, the solution became clear again.

The AuNRs covered with DNA were purified from free DNA strands through centrifugation. At least 4 centrifugation steps were used (6000 rcf, 20 min). Each time, the supernatant was carefully removed and the AuNRs were re-suspended in 0.5x TBE buffer containing 0.03% of SDS. The final concentration of the AuNRs was estimated with UV-Vis absorption spectroscopy using extinction coefficient of $9.5x10^8$ $M^{-1}cm^{-1}$ for the longitudinal plasmon resonance of the AuNRs (see Supplementary Fig. 15). We used AuNRs with dimensions of 10 nm x 38 nm as specified by supplier (Sigma-Aldrich, cat no. 716812). By utilization of AuNRs of smaller sizes or silver enhancement[8], the CD response of our plasmonic nanostructures can be shifted to shorter wavelengths.

Assembly of the AuNRs on the DNA origami template structures

The purified AuNRs were added to the purified DNA origami template structures in an excess of 10 AuNRs per DNA origami structure. The mixture was annealed from 40°C to 20°C over 15 hours. Additional annealing was crucial for achieving high yield of AuNR assembly. In contrast to our previous work[9], we used slightly longer AuNR capture strands (A_{10} instead of A_8) on DNA origami structures in order to ensure stable attachment of nanorods at 40°C used during CD characterization.

After thermal annealing, second agarose gel purification step (0.5% agarose gel in 0.5x TBE buffer with 11 mM $MgCl_2$) was used to separate the origami template structures assembled with two AuNRs from the AuNR excess as well as agglomerates containing a higher number of AuNRs and origami template structures. As shown in Supplementary Fig. 13, the band of the AuNR dimers on DNA origami was nicely separated from other products. This band was cut out from the gel. Then the structures were extracted with Freeze `N' Squeeze spin columns (BioRad) and used in CD measurements.

UV-Vis and CD characterization

CD and UV-Vis characterization was performed with a J-815 Circular Dichroism Spectrometer (Jasco) using Quartz SUPRASIL cuvettes (105.203-QS, Hellma Analytics) with a path length of 10 mm. Complementary UV-Vis characterization was performed with BioSpectrometer (Eppendorf).

In general, the CD signal increases linearly with the sample concentration. To obtain a good signal to noise ratio, we performed the measurements at concentrations corresponding to absorption values in the range of 0.2 – 0.5 at the longitudinal plasmonic resonance.

For CD characterization of the conformational switching, a 80 µL solution containing ~0.2 nM of the AuNR dimers was used. The absorption was ~0.4 at the longitudinal plasmon resonance. To ensure good switching efficiency, reliable functionality of the DNA assemblies and simultaneously avoid origami damage, the sample was kept at a temperature of 40 °C and pH 8 during the CD characterization. During the CD measurements, the cuvette was closed with a transparent lid to prevent solution evaporation.

Experiments on monitoring the conformational switching (Fig. 3d in the main text) were performed in the following way. The CD spectrometer was set to the time scan acquisition mode with a monitoring wavelength of 720 nm and a data pitch of 1 second. During UV/VIS light illumination the shutter of the CD spectrometer was closed. CD levels were measured for about 10 seconds with light UV /VIS illumination being switched off. Data points in Fig. 3d in the main text represent the mean value of measured CD.

Reversibility of the conformational switching (Fig. 4b in the main text and Supplementary Fig. 16 c,f) was examined by alternative UV and VIS light illumination in cycles for 15 and 10 min per exposure, respectively. After illumination, the CD signal was measured at 720 nm for about 1 min using the time scan acquisition mode of the CD spectrometer. Data points in Fig. 4b in the main text and Supplementary Figs. 16c,f represent the mean value of measured CD.

Theoretical calculations

Theoretical calculations were performed using commercial software COMSOL Multiphysics based on a finite element method. The origin of the bisignate CD in the plasmonic cross configuration is a consequence of interaction between the plasmons excited in the two AuNRs[10,11]. The CD signal was calculated as a difference in extinction for the left- and right-circularly polarized light. Since the plasmonic assemblies were dispersed in a solution, we carried out orientational averaging[12]. Averaging over all possible orientations at defined light incidence is equivalent to averaging over all incident directions of light for a nanostructure with defined orientation. It has been demonstrated both analytically and numerically[13,14] that averaging over six orthogonal directions of light incidence is sufficient to give accurate CD. In order to account for the inhomogeneous broadening due to the polydispersity of the AuNRs, the experimental dielectric function of Au was modified by including an additional term:

$$\varepsilon_{\text{effective}}(\omega)=\varepsilon_{\text{bulk}}(\omega)+\varepsilon_{\text{correction}}(\omega)\,, \qquad (1)$$

where the dielectric function of bulk Au, $\varepsilon_{\text{bulk}}$ is from Ref. 15 , and the correction term is introduced following a standard approach[16]

$$\varepsilon_{\text{correction}}(\omega)=\frac{\omega_{\text{p}}^2}{\omega^2+i\omega\gamma}-\frac{\omega_{\text{p}}^2}{\omega^2+i\omega\Gamma_{\text{broad}}}\,. \qquad (2)$$

The ω_p = 8.754 *eV* and γ = 0.0724 *eV* are the Drude parameters, respectively. Γ_{broad} is 0.290 *eV*.

Supplementary References

1. Douglas, S. M. *et al.* Rapid prototyping of 3D DNA-origami shapes with caDNAno. *Nucleic Acids Res.* **37,** 5001–5006 (2009).
2. Endo, M., Yang, Y., Suzuki, Y., Hidaka, K. & Sugiyama, H. Single-molecule visualization of the hybridization and dissociation of photoresponsive oligonucleotides and their reversible switching behavior in a DNA nanostructure. *Angew. Chem. Int. Ed.* **51,** 10518–10522 (2012).
3. Yang, Y., Endo, M., Hidaka, K. & Sugiyama, H. Photo-controllable DNA origami nanostructures assembling into predesigned multiorientational patterns. *J. Am. Chem. Soc.* **134,** 20645–20653 (2012).
4. Liang, X., Mochizuki, T. & Asanuma, H. A supra-photoswitch involving sandwiched DNA base pairs and azobenzenes for light-driven nanostructures and nanodevices. *Small* **5,** 1761–1768 (2009).
5. Kamiya, Y. & Asanuma, H. Light-driven DNA nanomachine with a photoresponsive molecular engine. *Accounts Chem. Res.* **47,** 1663–1672 (2014).

6. Zhang, X., Servos, M. R. & Liu, J. Instantaneous and quantitative functionalization of gold nanoparticles with thiolated DNA using a pH-assisted and surfactant-free route. *J. Am. Chem. Soc.* **134,** 7266–7269 (2012).
7. Shi, D., Song, C., Jiang, Q., Wang, Z.-G. & Ding, B. A facile and efficient method to modify gold nanorods with thiolated DNA at a low pH value. *Chem. Commun.* **49,** 2533–2535 (2013).
8. Kuzyk, A. *et al.* DNA-based self-assembly of chiral plasmonic nanostructures with tailored optical response. *Nature* **483,** 311–314 (2012).
9. Kuzyk, A. *et al.* Reconfigurable 3D plasmonic metamolecules. *Nat. Mater.* **13,** 862–866 (2014).
10. Auguié, B., Alonso-Gómez, J. L., Guerrero-Martínez, A. & Liz-Marzán, L. M. Fingers crossed: optical activity of a chiral dimer of plasmonic nanorods. *J. Phys. Chem. Lett.* **2,** 846–851 (2011).
11. Guerrero-Martínez, A. *et al.* Intense optical activity from three-dimensional chiral ordering of plasmonic nanoantennas. *Angew. Chem. Int. Ed.* **50,** 5499–5503 (2011).
12. Fan, Z. & Govorov, A. O. Plasmonic circular dichroism of chiral metal nanoparticle assemblies. *Nano Lett.* **10,** 2580–2587 (2010).
13. Fan, Z. & Govorov, A. O. Chiral nanocrystals: plasmonic spectra and circular dichroism. *Nano Lett.* **12,** 3283–3289 (2012).
14. Fasman, G. D. *Circular dichroism and the conformational analysis of biomolecules*. (Springer, 1996).
15. Johnson, P. B. & Christy, R. W. Optical constants of the noble metals. *Phys. Rev. B* **6,** 4370–4379 (1972).
16. Kreibig, U. & Vollmer, M. *Optical properties of metal clusters*. (Springer, 1995).
